# Structural insights into the mechanism of leptin receptor activation

**DOI:** 10.1038/s41467-023-37169-6

**Published:** 2023-03-31

**Authors:** Robert A. Saxton, Nathanael A. Caveney, Maria Dolores Moya-Garzon, Karsten D. Householder, Grayson E. Rodriguez, Kylie A. Burdsall, Jonathan Z. Long, K. Christopher Garcia

**Affiliations:** 1 Department of Molecular and Cellular Physiology, Stanford University School of Medicine, Stanford, CA 94305 USA; 2 Department of Pathology, Stanford University School of Medicine, Sarafan ChEM-H, Stanford University, Stanford, CA USA; 3 Program in Immunology, Stanford University School of Medicine, Stanford, CA 94305 USA; 4 Cancer Biology Program, Stanford University School of Medicine, Stanford, CA 94305 USA; 5 Department of Structural Biology, Stanford University School of Medicine, Stanford, CA 94305 USA; 6 Howard Hughes Medical Institute, Stanford University School of Medicine, Stanford, CA 94305 USA; 7 Present Address: Department of Molecular and Cell Biology, University of California at Berkeley, Berkeley, CA 94720 USA

**Keywords:** Hormones, Cryoelectron microscopy, Permeation and transport

## Abstract

Leptin is an adipocyte-derived protein hormone that promotes satiety and energy homeostasis by activating the leptin receptor (LepR)–STAT3 signaling axis in a subset of hypothalamic neurons. Leptin signaling is dysregulated in obesity, however, where appetite remains elevated despite high levels of circulating leptin. To gain insight into the mechanism of leptin receptor activation, here we determine the structure of a stabilized leptin-bound LepR signaling complex using single particle cryo-EM. The structure reveals an asymmetric architecture in which a single leptin induces LepR dimerization via two distinct receptor-binding sites. Analysis of the leptin–LepR binding interfaces reveals the molecular basis for human obesity-associated mutations. Structure-based design of leptin variants that destabilize the asymmetric LepR dimer yield both partial and biased agonists that partially suppress STAT3 activation in the presence of wild-type leptin and decouple activation of STAT3 from LepR negative regulators. Together, these results reveal the structural basis for LepR activation and provide insights into the differential plasticity of signaling pathways downstream of LepR.

## Abstract (web summary)

Leptin is a master regulator of body weight and energy homeostasis that is dysregulated in obesity. Here, authors determined the cryo-EM structure of the activated Leptin receptor complex, revealing insights into the mechanism of Leptin signaling

## Introduction

Excess body weight is a major underlying risk factor for a variety of human diseases including type 2 diabetes, cardiovascular disease, and many types of cancer^1^. Over 30% of adults and 20% of adolescents in the United States are now categorized as obese, with prevalence increasing steadily^2^. Although a variety of dietary and pharmacological interventions have emerged in the past several decades to combat obesity, therapeutics capable of safely promoting significant and sustained weight loss are still needed^3^.

Leptin is an adipocyte-derived protein hormone discovered in the 1990s as a critical regulator of body weight in mammals^4,5^. Genetic loss of leptin results in increased food intake and severe early onset obesity in both mice and humans, and administration of recombinant leptin is sufficient to restore normal body weight in this context^6–9^. Although these observations led to substantial interest in the clinical use of leptin for obesity, exogenous leptin treatment is not effective in most obese patients, nearly all of whom already exhibit significantly elevated plasma leptin levels but diminished leptin responsiveness^10–12^.

Leptin exerts its satiety-promoting effects by activating the leptin receptor (LepR) on the surface of a subset of hypothalamic neurons^5,13^. Activation of LepR in turn drives the phosphorylation and activation of the transcription factor Signal Transducer and Activator of Transcription 3 (STAT3), which drives production of anorexigenic peptides that suppress food intake and increase energy expenditure. Physiologically, leptin is produced by adipocytes such that levels of circulating leptin are proportional to the amount of adipose tissue, thereby serving as a homeostatic feedback mechanism to correlate food intake with organismal energy stores^5,14^. In the context of obesity, however, this pathway is dysregulated, resulting in a failure to reduce food intake despite excess adiposity and high levels of circulating leptin^11,12^. Although the mechanism of this observed leptin resistance is not fully understood, evidence suggests that it is results from chronic hyperleptinemia and the resulting desensitization of LepR to leptin, due in part to the expression and recruitment of cytosolic LepR antagonists such as Suppressor of Cytokine Signaling 3 (SOCS3)^15–20^.

LepR is a member of the class 1 cytokine receptor family which includes the related receptors glycoprotein 130 (gp130) and LIF receptor (LIFR)^1,2^. Previous biochemical and mutagenesis studies have identified two distinct leptin-binding regions within LepR, including a high affinity “site 2”, within the second cytokine homology region (CHR2) of LepR^21^, and a low affinity “site 3” within the immunoglobulin (Ig) domain^22^. Previous attempts to resolve the structure of leptin-bound LepR have been limited to low resolution (~40 Å), due in part to the instability of the 2:2 leptin–LepR complex in vitro^23^. Here we determine the cryo-EM structure of a stabilized leptin–LepR signaling complex, revealing new insights into the mechanism of LepR activation and potential avenues for the pharmacological modulation of leptin signaling.

## Results

### Cryo-EM structure of the leptin receptor signaling complex

**Fig. 1:**
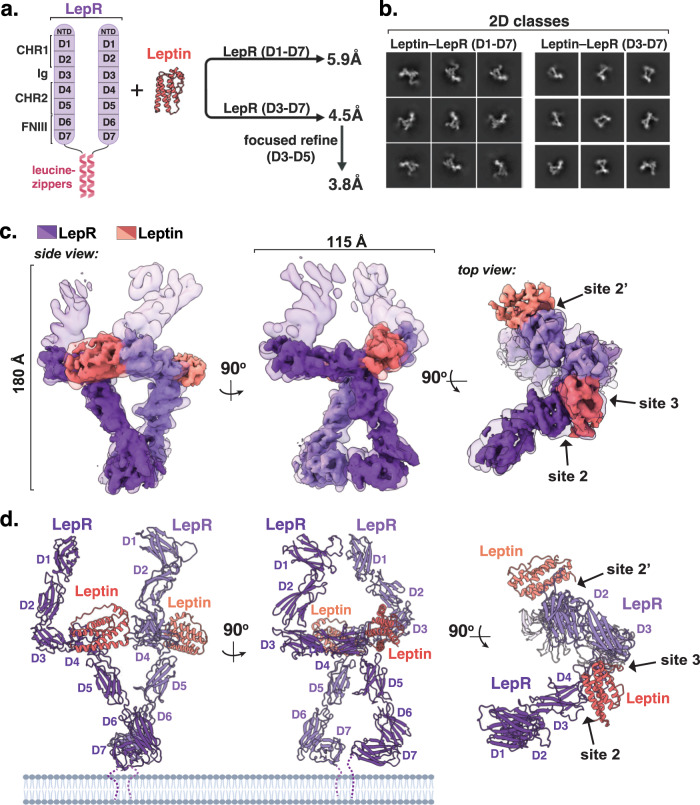
Cryo-EM structure of the leptin receptor signaling complex. **a** Schematic depicting LepR ECD domain architectures, and the regions analyzed for structure determination using cryo-EM. **b** Cryo-EM 2-dimensional class averages of assembled leptin-bound LepR^D1–^^D7^ and LepR^D3–^^D7^ complexes. **c** Overlaid segmented density maps of the Leptin-LepR^D1–^^D7^ and Leptin-LepR^D3–^^D7^ complexes resolved to 5.9 Å and 4.5 Å resolution, respectively. **d** Three views of the leptin-LepR^D1–^^D7^ structural model, with leptin in salmon and LepR in purple.

To gain insight into the mechanisms of leptin receptor activation, we sought to determine the structure of the active LepR signaling complex. To stabilize the leptin–LepR complex, we engineered a construct comprising the complete extracellular domain (D1–D7) of LepR with the transmembrane (TM) domains replaced by dimerizing leucine zippers (LepR^D1–^^D7^-zip), thereby enhancing the avidity of LepR for leptin (Fig. 1a–d). Recombinantly expressed mouse LepR^D1–^^D7^-zip formed a stable complex with mouse leptin in vitro as assessed by co-elution over size-exclusion chromatography (Supplementary Fig. 1a–d). Analysis of this complex using single particle cryo-electron microscopy (cryo-EM) yielded a 3D reconstruction of the leptin–LepR^D1-D7^ complex to 5.9 Å resolution (Fig. 1a, b; Supplementary Fig. 2a–e). Given that flexibility of the membrane distal D1 and D2 domains of LepR appeared to limit the resolution of these reconstructions, we next purified and performed cryo-EM analysis on a truncated leptin–LepR^D3–^^D7^-zip complex, which yielded an improved 4.5 Å resolution 3D reconstruction (Fig. 1a, b; Supplementary Fig. 3a–i). Using AlphaFold models^24^ of mouse leptin and monomeric LepR, these maps enabled high confidence docking and refinement of the complete leptin–LepR signaling complex (Fig. 1c, Supplementary Fig. 4a–d).

Analysis of the resulting molecular model of the 2:2 leptin–LepR complex unexpectedly revealed an asymmetric, partially open conformation in which one leptin simultaneously binds both LepR chains, whereas the second leptin engages only a single LepR subunit (Fig. 1c). Consistent with previous mutagenesis studies^21^, the high affinity leptin–LepR interaction, referred to as “site 2,” is formed by the hinge of the LepR CHR2 (domains D4–D5), which engages helices A and C of leptin (Fig. 1c). The low affinity site 3 interface by contrast is formed by the immunoglobulin (Ig) domain of LepR (D3)^22^, which interacts with the top of leptin helix D and loop AB. Notably, the canonical “site 1” interface, which participates in high affinity receptor binding in most cytokine receptor complexes^25^, is unoccupied in the leptin–LepR complex (Fig. 1d).

Above the leptin-binding regions, the membrane distal CHR1 (D1 and D2 domains) bend upwards, resulting in a highly elongated structure with a total estimated length of approximately 180 Å projecting from the cell surface (Fig. 1c, d). Below the leptin-binding interface, the two-membrane proximal fibronectin domains (FNIII, D6 and D7) of LepR bend inwards towards one another, forming an approximately 90^o^ angle between D6 and D7 which is predicted to bring to TM domains of each LepR monomer into proximity. Notably, there is no direct interaction between the two LepR ECDs, suggesting that receptor dimerization is primarily ligand-dependent, as has been observed for other class 1 cytokine receptors^26^.

### Structural homology between leptin and IL-6 family cytokine receptor complexes

**Fig. 2:**
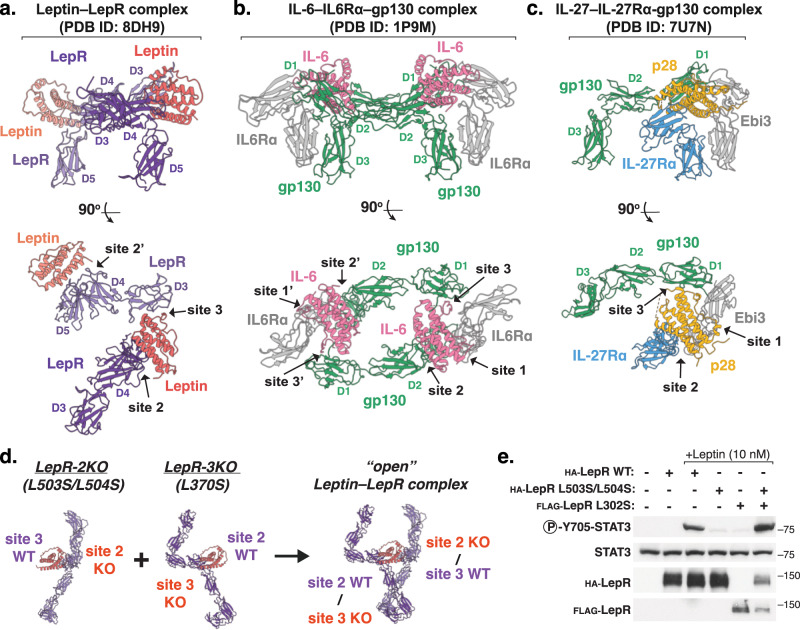
Structural homology between leptin and IL-6 family cytokine receptor complexes. **a** Side and top views of leptin-bound LepR structural model (PDB ID: 8DH9), showing the leptin-binding domains (D3-D5) of LepR, with leptin in salmon and LepR in purple. **b** Side and top views of the hexameric IL-6 receptor complex (PDB ID: 1P9M) showing IL-6 in pink, gp130 in green, and IL-6Rα in gray. **c** Side and top views of the IL-27 receptor complex (PDB ID: 7U7N), showing IL-27 subunits p28 and Ebi3 in yellow and gray, respectively, gp130 in green, and IL-27Rα in blue. **d** Schematic showing how LepR mutants L503S/L504S (LepR-2KO) and L370S (LepR-3KO) assemble to exclusively form an asymmetric 1:2 leptin–LepR complex, in the same conformation as the partially open 2:2 complex observed in our structure. **e** Immunoblot of lysates prepared from HEK-293T cells transiently expressing the indicated LepR constructs and stimulated with 10 nM recombinant leptin for 20 min. Representative result of experiment performed three times.

Based on both sequence and structural homology, leptin is most closely related to the IL-6 family cytokines, which exert diverse biological effects through the shared receptor gp130 (*29*) (Fig. 2a–c). Comparison of the cryo-EM structure of leptin–LepR reported here with the hexameric complex of IL-6 bound to IL-6Rα and gp130 reveals that the docking modes of the site 2 and site 3 interactions between leptin and LepR are similar to those formed between IL-6 and gp130 (*30*) (Fig. 2a, b). However, in the IL-6 structure, the Ig domains of both gp130 subunits bend back in to engage the adjacent IL-6 molecules, forming two site 3 interactions in a symmetric, closed receptor conformation (Fig. 2b). By contrast, the Ig domain of only a single LepR engages leptin at site 3, whereas the Ig domain of the second LepR projects outward, away from the second bound leptin which is unoccupied at site 3 (Fig. 2a). As a result, despite being a 2:2 homodimeric receptor complex, the architecture of the leptin-bound LepR complex more closely resembles heterodimeric IL-6 family receptor complexes, such as IL-27, in which a single ligand dimerizes two different receptors, gp130 and IL-27Rα, to form a similarly open and asymmetric receptor complex^27^ (Fig. 2c).

The asymmetric conformation observed in our structure suggests that a single leptin molecule is sufficient to dimerize two LepR chains and activate downstream STAT3 signaling. To test this hypothesis, we transfected HEK-293T cells with cDNA encoding either WT LepR, LepR lacking site 2 binding^21^ (LepR-2KO, L503S/L504S), and/or LepR lacking site 3 binding^22^ (LepR-3KO, L370S, Fig. 2d). Whereas cells expressing LepR-2KO or LepR-3KO alone failed to respond to recombinant leptin, cells co-expressing both mutant receptors exhibited full leptin responsiveness, as assessed by phosphorylation of STAT3 (Fig. 2e, Supplementary Fig. 5a). Given that cells co-expressing these two receptor mutants can only engage leptin in an “open” 1:2 orientation (Fig. 2d), these data demonstrate that the asymmetric conformation observed in our structures represents an active signaling complex. Moreover, computationally modeling a hypothetical “closed” LepR complex by rotating the Ig domain of the second LepR results in a predicted clash between the membrane proximal D7 domains of the two LepR chains (Supplementary Fig 5b, c). Thus, although we cannot rule out the formation of a closed LepR complex on the cell surface, perhaps transiently or in a dynamic equilibrium with the asymmetric complex, this would require significant rearrangement of LepR domains compared to what is observed in our structures.

### Structural basis for leptin-dependent LepR dimerization

**Fig. 3:**
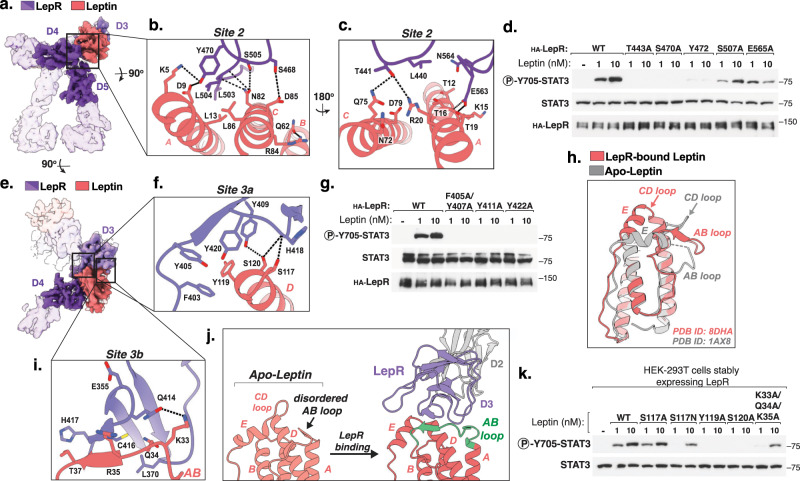
Structural basis for leptin-dependent LepR dimerization. **a** Front view of the segmented density map of the leptin-LepR^D3-D7^ complex resolved to 4.5 Å resolution (transparent) with the focus refined map encompassing leptin and the leptin-binding domains of LepR, resolved to 3.8 Å resolution (solid). **b**, **c** Close-up views of the leptin-LepR site 2 binding interface. Hydrogen bonds and salt-bridges are shown as black dashed-lines. **d** Immunoblot of lysates prepared from HEK-293T cells transiently expressing the indicated LepR constructs and stimulated with the indicated concentration of recombinant leptin for 20 min. Representative result of experiment performed two times. **e** Top view of the segmented density maps shown in (**a**). **f** Close-up view of the leptin-LepR site 3a binding interface. **g** Immunoblot of lysates prepared and analyzed as in (**c**). Representative result of experiment performed two times. **h** Comparison of the apo-leptin structure (PDB ID: 1AX8) and LepR-bound leptin structure (this paper, PDB ID: 8DHA), showing leptin in salmon, LepR D3 in purple. **i** Close-up view of the leptin-LepR site 3b binding interface. **j** LepR-dependent ordering of the leptin AB loop residues 24–39, shown in green. **k** Immunoblot of lysates prepared from HEK-293T cells stably expressing wild-type LepR and stimulated with the indicated leptin variants for 20 min. Representative result of experiment performed three times.

Focused refinement of the LepR^D3-D7^-zip complex centered on the leptin–LepR interface yielded an additional 3.8 Å resolution map comprising leptin together with the three interacting domains of LepR (D3 and D4/D5), enabling molecular analysis of the site 2 and site 3 binding interfaces (Fig. 3a and Supplementary Fig. 3g–i). At the high affinity site 2, the A and C helices of leptin engage several loops in the CHR2 (D4 and D5) of LepR, burying 750 Å^2^ of surface area (Fig. 3b, c). The interaction appears to be mediated in large part by hydrophobic contacts, such as between Leu13 and Leu86 of leptin and Leu503 and Leu504 of LepR (Fig. 3b). This is consistent with previous reports that mutation of Leu503 and Leu504 in LepR abolishes leptin responsiveness^28^ (Fig. 2e). In addition, several apparent polar and electrostatic contacts are also formed between leptin and LepR at this interface, including between leptin residues Asp9, Thr16, and Asp85, which appear to contact LepR residues Tyr470, Glu563, and Ser468, respectively. Moreover, leptin residues Arg20 and Gln75, which were previously reported to be required for LepR binding^29^, form apparent hydrogen bonds with Thr441 in LepR (Fig. 3c). Consistent with this, mutation of the corresponding site 2 contact residues in human LepR to alanine (T443A, S470A, and Y472A, and E565A) resulted in substantially reduced leptin responsiveness as assessed by phosphorylation of STAT3 in HEK-293T cells (Fig. 3d, Supplementary Fig. 7a).

At the low affinity site 3 interface, the Ig domain of LepR (D3) engages the top of helix D and the CD loop of leptin (Fig. 3d, e), burying 775 Å^2^. The primary contact appears to be mediated by Tyr119 within the CD loop of leptin, which inserts into an aromatic cluster consisting of mouse LepR residues Phe403, Tyr405, Tyr409, and Tyr420 (“site 3a,” Fig. 3e, f). Consistent with a key role for these aromatic contacts in LepR activation, mutation of the corresponding aromatic residues in human LepR including F405A/Y407A, Y411A, or Y422A, all yielded diminished leptin-dependent signaling (Fig. 3g, Supplementary Fig. 7b). Immediately adjacent to Tyr119, leptin residue Ser120 forms apparent hydrogen bonds with both the hydroxyl of Tyr409 and the backbone carbonyl of His418, which is also contacted by the neighboring leptin residue Ser117 (Fig. 3f).

Comparison of the previously reported apo-leptin structure with the LepR-bound structure reported here reveals a substantial rearrangement of leptin helix E as well as loops AB and CD loops (Fig. 3h). This results in large part from a second site 3 contact site formed between the leptin AB loop and the LepR Ig domain via a primarily backbone-mediated β-sheet interaction formed between LepR residues 415–417 and leptin residues 35–37 (“site 3b”, Fig. 3g). The side chains of neighboring leptin residues Gln34 and Lys33 appear also form additional contacts with Cys416 and Gln414 of LepR, respectively (Fig. 3i). Notably, a large portion of the leptin AB loop (residues 24–39), including the region implicated in the site 3B interaction, is disordered in the crystal structure apo-leptin, but ordered upon LepR binding (Fig. 3j). LepR binding therefore appears to induce a large conformational change in leptin that stabilizes the site 3 interaction and facilitates LepR dimerization.

To assess the functional importance of the leptin residues implicated in site 3 binding in our structure, we expressed and purified human leptin variants with mutations at key site 3 interaction residues and assessed their effect on LepR signaling in HEK-293T cells expressing LepR (Supplementary Fig. 7c). Notably, mutation of site 3a residues Tyr119 or Ser120 to alanine (Y119A and S120A) abolished leptin signaling activity as assessed by phosphorylation of STAT3 (Fig. 3k). Mutation of the neighboring Ser117 to Ala (S117A) however had minimal impact on LepR signaling, although mutation to Asn (S117N) resulted in partially reduced STAT3 activation, likely by creating a steric clash with His418 of LepR (Fig. 3f). Simultaneous mutation of three site 3b contact residues in the AB loop of leptin (K33A/Q34A/K35A, corresponding to mouse residues K33/Q34/R35) similarly resulted in partially reduced STAT3 activation (Fig. 3k, Supplementary Fig. 7d, e). Together, these results suggest that leptin-mediated LepR dimerization is primarily driven by the aromatic and polar contacts formed by leptin residues Tyr119 and S120 at site 3a, with the receptor-induced conformational change of the leptin AB loop playing a secondary role to further stabilize the LepR dimer and enable maximal STAT3 activation.

Analysis of the site 2 and site 3 leptin–LepR interfaces also provides insight into the molecular basis of several human obesity-associated mutations in leptin, including *D79Y*, *N82K*, *R84W*, and *S120C* (Fig. 3b, c, f)^21^. Notably, Asn82 of leptin lies in near the center of the site 2 interface and makes key hydrogen bonds contacts with LepR residues Ser505 and Leu503, suggesting a loss of site 2 binding associated with the *N82K* mutation (Fig. 3b). The neighboring residues Asp79 and Arg84 are also near the site 2 interface, but do not directly contact LepR, instead making apparent intramolecular contacts with leptin residues Arg20 and Gln62 (Fig. 3b, c), likely indirectly stabilizing the site 2 interaction. Although most obesity-associated mutations in LepR are distal to the leptin-binding sites and likely act to simply destabilize LepR, two previously identified human obesity-associated LepR mutations (*A409E* and *Y422H*)^22,23^ both occur at the site 3 binding interface and are predicted by our structure to disrupt the key aromatic contacts formed by leptin Tyr119 (Fig. 3f).

### Biased leptin analogs decouple activation of STAT3 from LepR negative regulators

**Fig. 4:**
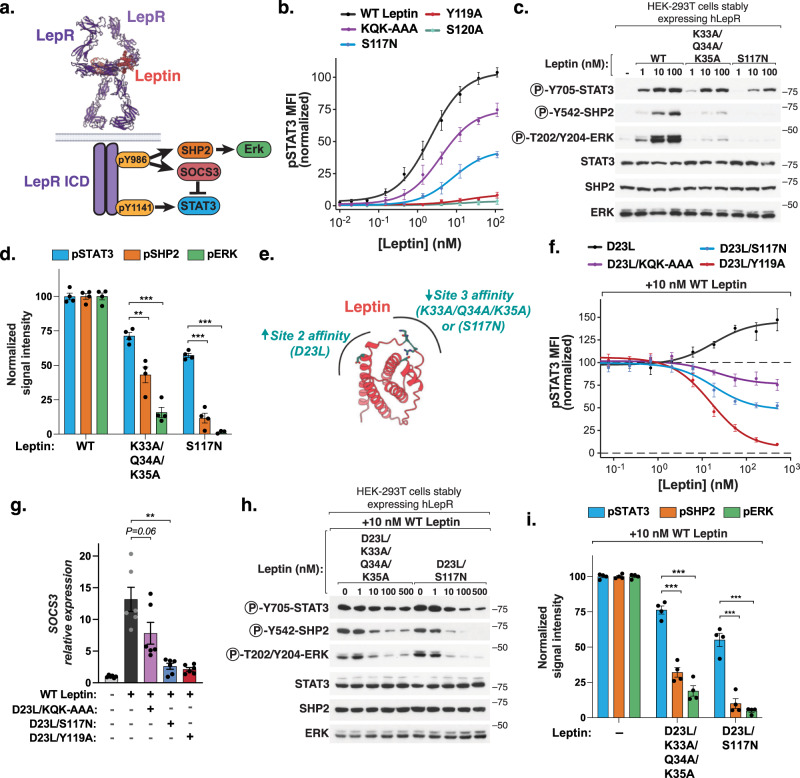
Biased leptin analogs decouple activation of STAT3 from LepR negative regulators. **a** Cartoon model of LepR signaling in which STAT3 recruited by phosphorylation of LepR-Y1141, whereas LepR negative regulators are recruited by phosphorylation of LepR-Y986. **b** Phospho-STAT3 dose-response curves for WT or mutant leptin in LepR-expressing HEK 293T cells, analyzed by flow cytometry and shown as a percent of maximal WT leptin mean fluorescent intensity (MFI ± SEM, *n* = 4 (mutants) or 6 (WT) independent replicates). **c** Immunoblot of lysates prepared from HEK-293T cells stably expressing wild-type LepR that were serum starved for 18 h and then stimulated with the indicated leptin variants for 20 min. Representative result of experiment performed four times. **d** Quantification of immunoblots prepared as in (**c**) (100 nM leptin, mean ± SEM, *n* = 4 independent replicates, *t*-test, **p = 0.004, ***P < 0.001). **e** Schematic showing engineering strategy to create high affinity LepR partial agonists, through increasing affinity of leptin for LepR at site 2 and decreasing affinity at site 3. **f** Phospho-STAT3 signaling in LepR-expressing HEK 293T cells stimulated with 10 nM WT Leptin and the indicated concentration of leptin variants, analyzed as in (B) (mean ± SEM, *n* = 4 independent replicates). **g** Relative expression of SOCS3 from hLepR-expressing HEK 293T cells stimulated with 10 nM WT Leptin and 100 nM of the indicated leptin variants for 6 h, analyzed by RT-qPCR. (mean ± SEM, *n* = 6 independent replicates, *t*-test, **p = 0.003). **h** Immunoblot of lysates prepared from HEK-293T cells stably expressing wild-type hLepR that were serum starved for 18 h and then stimulated with 10 nM WT leptin and the indicated concentration of the indicated leptin variants for 20 min. Representative result of experiment performed four times. **i** Quantif**i**cation of immunoblots prepared as in (**h**). Cells treated with 100 nM leptin variants (± SEM, *n* = 4 independent replicates, *t*-test, ***p < 0.001).

The leptin-mediated dimerization of LepR results in the JAK2-dependent phosphorylation of multiple tyrosine residues on the intracellular domain (ICD) of LepR, each of which have distinct biological roles^5,13^. Specifically, phosphorylation of Tyr1141 (Y1138 in mice) drives activation of STAT3 and the subsequent satiety promoting effects of leptin, whereas phosphorylation of Tyr986 (Tyr 985 in mice) activates the SHP2/ERK pathway and is required for recruitment of cytosolic LepR antagonists that drive leptin resistance^5,13,24,25^ (Fig. 4a). Consistent with this, mice expressing a Y1138S LepR mutant are extremely obese and hyperphagic due to loss of leptin-mediated STAT3 activation^26,30^. By contrast, mice expressing a Y985F LepR mutant are lean and do not become leptin resistant, phenocopying loss of hypothalamic SOCS3 expression^15,27,31^.

Previously, we showed that partial agonists that destabilize assembly of the heterodimeric cytokine receptor IL-22R elicited biased signaling by inducing differential tyrosine phosphorylation on the receptor ICD^32^. Given the unexpected asymmetric nature of the leptin-LepR complex observed in our structure, we hypothesized that a similar approach could be used to decouple activation of Y986/SHP2/ERK pathway from the stimulation of Y1141/STAT3 downstream of LepR. To this end, we first assessed whether various leptin site 3 mutants exhibited partial agonism (i.e., sub-maximal signaling at saturating ligand concentrations), by performing comprehensive STAT3 dose-response analysis using phospho-flow cytometry. The human leptin mutants K33A/Q34A/K35A (KQK-AAA) and S117N displayed partial agonism of human LepR, with maximal STAT3 responses of approximately 75% and 45% of WT leptin, respectively (Fig. 4b, Supplementary Fig. 7f). Remarkably, both of these variants also elicited biased agonism, with preferential activation of STAT3 relative to SHP2 or ERK when compared to WT leptin (Fig. 4c, d). Similarly, in cells expressing mouse LepR, the partial agonist variant S117A elicited approximately 60% activation of STAT3 but selectively weaker activation of both SHP2 (20%) and ERK (5%) (Supplementary Figs. 7g, h and 8a–c). This level of partial STAT3 agonism was nonetheless sufficient to promote satiety and weight loss in leptin-deficient obese (ob/ob) mice (Supplementary Fig. 8d, e). Together, these data suggest that partial destabilization of leptin-mediated LepR dimerization via modulation of the site 3 binding interface results in STAT3-biased agonism in both mouse and human LepR-expressing cells.

An important consideration for the development of therapeutic LepR agonists is that the serum levels of endogenous leptin are highly elevated in the context of human obesity, ranging from 5 ng/ml (0.3 nM) in lean individuals to as high as 100 ng/ml (6 nM) in obesity^33,34^. We therefore assessed whether the LepR partial agonists KQK-AAA and S117N could modulate downstream LepR signaling in the presence of high levels of WT leptin. To enhance the ability of these variants to compete with WT leptin for LepR binding, we incorporated an additional mutation at site 2, *D23L*, which reduces the off rate of leptin for LepR^35^ (Fig. 4e). These high affinity partial agonists enforced sub-maximal STAT3 activation and a corresponding reduction in SOCS3 expression even in the presence of 10 nM WT leptin, thereby functioning as “receptor signaling clamps”^36^ (Fig. 4f, i). Moreover, these variants suppressed phosphorylation of SHP2 and ERK to a significantly greater extent than STAT3, with the D23L/S117N variant in particular reducing both SHP2 and ERK signaling by approximately 90%, despite retaining approximately 50% STAT3 activation relative to treatment with WT leptin alone (Fig. 4g, h). Thus, high affinity LepR biased agonists decouple activation of STAT3 from the Tyr986/SHP2/ERK signaling axis, even in the presence of WT leptin.

## Discussion

Together with previous biochemical studies^27,32^, the cryo-EM structures reported here support a two-step mechanism of LepR activation in which leptin first binds LepR via the high affinity site 2 interface to form a 1:1 complex on the cell membrane. Subsequent trans-interaction between leptin and the LepR Ig domain forms the site 3 interface, which dimerizes LepR to form signaling competent 2:2 complexes. Surprisingly, our cryo-EM data reveal that a partially open, asymmetric 2:2 complex in which only one leptin forms a site 3 contact with LepR is the most stable dimer conformation in solution. This asymmetric receptor homodimerization is reminiscent of the asymmetry seen in some homodimeric receptor tyrosine kinases such as Insulin receptor and Insulin-like Growth Factor 1 receptor (IGF-1R)^37–39^, but is unique among known cytokine receptor complexes. Given the extremely low affinity of the leptin–LepR site 3 interaction, it is likely that the complex dynamically interchanges between 1:1 and partially open 2:2 complexes, with minimal sampling of the fully closed 2:2 conformation.

The asymmetric conformation observed here is distinct from previous modeling of the 2:2 leptin–LepR complex based on a low resolution (40 Å) negative stain EM data^23^. Interestingly, the authors in that study observed that most quaternary complex projections in their dataset were asymmetric, but these were attributed distortion from the use of a carbon support and discarded^23^. In our dataset, no symmetric 2:2 particle classes were observed.

The open LepR conformation observed in our structure suggests the potential for the formation of higher order leptin–LepR assemblies. While this manuscript was in review, the structure of a 3:3 leptin–LepR complex was reported^40^. Although this closed 3:3 complex is compatible with the open 2:2 structure reported here, our data also show that mutant LepR complexes that can only form asymmetric LepR dimers, but not trimers, exhibit full signaling activity in cultured cells (Fig. 2e), consistent with JAK2 homodimerization driving downstream LepR signaling^5,13,41,42^. Moreover, engineered antibodies that induce LepR homodimerization independently of leptin have been shown to exert full agonist activity, suggesting recruitment of two copies of LepR is sufficient for full receptor activation^43^. The open 2:2 architecture observed here therefore likely represents the minimal fully active LepR signaling complex, and the functional role of the symmetric 3:3 assembly remains to be determined.

Consistent with the site 3 leptin–LepR interaction being the rate-limiting step for leptin-dependent signaling, our mutagenesis data reveal that the modulation of the site 3 binding affinity can tune LepR signal strength across a wide dynamic range (Fig. 4b). Moreover, some leptin site 3 mutants also exhibited biased agonism, selectively promoting signaling through the LepR^Tyr1141^–STAT3 axis with diminished activation of the LepR^Tyr986^–SHP2–ERK axis. Although we are unable to directly assess site-specific LepR ICD phosphorylation with available reagents, these results indicate that phosphorylation of Tyr1141 may occur more efficiently than phosphorylation of Tyr986, such that reducing stability of the 2:2 complex more substantially impacts phosphorylation at Tyr986. Alternatively, the phospho-tyrosine binding domains of STAT3 and SHP2 may have different affinities for each LepR phospho-tyrosine, such that even uniform reduction of LepR ICD phosphorylation may differentially impact these two pathways. Distinguishing between these and other possible mechanisms will require further biochemical analysis of the LepR ICD interactions and may be informative for future development of biased LepR agonists.

## Methods

### Protein production and purification

For structural studies, mouse LepR^D1-D7^ (*Mus musculus*, residues 23–839) and LepR^D3-D7^ (*M. musculus*, residues 330–839) were cloned into a pD649 mammalian expression vector containing an N-terminal HA signal peptide, C-terminal GCN4 leucine zipper (EELLSKNYHLENEVARLKK), and C-terminal 6xHis-tag. DNA was transiently transfected into Expi293F cells (Thermo, A14528) using Expifectamine transfection reagent (Thermo). Expi293F cells were grown in serum free Expi293 expression media (Thermo) and maintained at 37 °C with 5% CO_2_ with gentle agitation. 72 h after transfection, cell supernatant was harvested and proteins were purified with Ni-NTA resin (Qiagen) followed by size-exclusion chromatography (SEC) on a Superdex 200 column (GE) in Phosphate-buffered saline (PBS, 135 mM NaCl, 2.5 mM KCl, 8.0 mM Na_2_HPO_4_, 30 mM KH_2_ PO_4_, pH 7.2).

Purified receptor complexes were incubated overnight at 4 °C with *Escherichia coli* produced *M. musculus* leptin with a leptin:LepR ratio of 4:1. The leptin–LepR complex was then re-purified by SEC on a Superdex 200 column in PBS. Purified sample was crosslinked with 1 mM bis(sulfosuccinimidyl)suberate (BS3) (Thermo) for 45 min at room temperature and quenched with 20 mM Tris-HCl pH 8. Crosslinked complex was re-purified by SEC on a Superdex 200 column in PBS and concentrated to 1–2 mg/ml for cryo-EM analysis.

For signaling experiments, wild-type or mutant leptin (*Homo sapiens*, residues 23–167) was cloned into a pET28a *E. coli* expression vector containing a C-terminal 6xHis-tag. DNA was transformed into BL21(DE3) competent cells and grown at 37 °C in LB media supplemented with Kanamycin (40 μg/mL) until the culture reached log-phase growth. Protein expression was induced by adding IPTG at a final concentration of 1 mM. The culture was shaken at 37 °C for 4 h. Cells were harvested by centrifugation at 6000 × *g* for 6 min, and frozen at −20 °C.

Cell pellets were resuspended in Lysis Buffer (50 mM Tris-HCl pH 8.0, 1% (v/v) TritonX-100, 100 mM NaCl, 5 mM MgCl_2_, 1 mg Benzonase) and lysed by sonication. Inclusion bodies were isolated by centrifugation at 10,000 × *g* for 15 min. Inclusion bodies were washed 3× in Wash Buffer 1 (50 mM Tris-HCl pH 8.0, 0.5% TritonX-100, 100 mM NaCl, 1 mM Na EDTA, 1 mM DTT) and 1× in Wash Buffer 2 (50 mM Tris-HCl pH 8.0, 100 mM NaCl, 1 mM Na EDTA, 1 mM DTT), with centrifugation at 10,000 × *g* for 15 min between each wash. Inclusion bodies were then solubilized by rotating in Denaturing Buffer (20 mM Tris-HCl pH 8.0, 8 M Urea, 1 mM DTT) at room temperature for 24 h. Solubilized samples were then centrifuged at 30,000 × *g* for 30 min, and supernatant was frozen at −80 for further processing.

Leptin proteins were refolded by dropwise addition into cold Refolding Buffer (100 mM Tris-HCl pH 8.0, 400 mM l-Arginine, 1 mM Oxidized Glutathione, and 10 mM reduced Glutathione) to a final concentration of approximately 0.05 mg/ml and stirred gently at 4 °C for 48 h. Samples were then filtered through a 0.22 μM Millipore Express© PLUS filter, concentrated 30-fold before being purified by SEC on a Superdex 75 column in Tris-buffered saline (TBS, 20 mM Tris-HCL pH 8.0, 150 mM NaCl). Proteins were maintained in TBS, concentrated to approximately 1 mg/mL before being flash-frozen in liquid nitrogen and stored at −80 °C for future use.

For in vivo studies, cDNAs encoding WT or mutant leptin were cloned into a pD649 mammalian expression vector containing an N-terminal HA signal peptide, N-terminal mosue-serum albumin (MSA) tag, and C-terminal 6xHis-tag. DNA was transiently transfected into Expi293F cells (Thermo) using Expifectamine transfection reagent (Thermo). Expi293F cells were grown in serum free Expi293 expression media (Thermo) and maintained at 37 °C with 5% CO_2_ with gentle agitation. Seventy two hours after transfection, cell supernatant was harvested and proteins were purified with Ni-NTA resin (Qiagen) followed by size-exclusion chromatography (SEC) as described above.

### Cryo-EM specimen preparation and data collection

Aliquots of 3 μL of the LepR^D1–^^D7^ and LepR^D3–^^D7^ receptor complex were supplemented with 0.01% fluorinated octyl maltoside (Anatrace) and immediately applied to glow-discharged Quantifoil® (1.2/1.3) grids. The grids were blotted for 3 s at 100% humidity with an offset of +3 and plunge frozen into liquid ethane using a Vitrobot Mark IV (Thermo Fisher). Grids were imaged on a 300 keV Titan Krios cryo-electron microscope (Thermo Fisher) equipped with a K3 camera (Gatan). Additionally, an energy filter (Gatan) was used during imaging of the LepR^D1-D7^ complex. Movies were collected at a calibrated magnification corresponding to 0.653 Å and 0.8521 Å per physical pixel, for LepR^D1–^^D7^ and LepR^D3–^^D7^ complexes, respectively. The dose was set to a total of 53 electrons per Å^2^ over an exposure of 1.518 and 2.545 seconds, for LepR^D1–^^D7^ and LepR^D3–^^D7^ complexes, respectively. Automated data collection was carried out using SerialEM with a nominal defocus range set from 0.8 to 2.0 μM. 11,292 movies were collected for the complex with LepR^D1–^^D7^ and 21,112 movies were collected for the complex with LepR^D3–^^D7^.

### Cryo-EM Data processing and 3D reconstruction

All movies were processed using cryoSPARC v3.1.0 (44) unless otherwise specified. Movies were motion corrected, had contrast transfer functions (CTFs) determined, and particles picked using the cryoSPARC live processing functions. During this processing, micrographs were binned to the physical pixel size.

For the LepR^D1–^^D7^ receptor complex, a prior test collection using no BS3 crosslinker in sample preparation was used in successive rounds of reference-free 2D classification, leaving 607,681 particles in well-defined classes. These particles were used to generate one good and two bad ab initio models. These ab initio models were then used in six rounds of iterative heterogenous refinement with 1,735,072 particles of the crosslinked data. This resulted in a class with 270,721 particles which had a resolution of 6.4 Å when refined with non-uniform refinement^44^. Local Refinement, with a generous mask around defined regions of density, was then used to generate a final map at 5.9 Å resolution, which was sharpened using LAFTER^45^.

For the LepR^D3–^^D7^ receptor complex, successive rounds of reference-free 2D classification of 17,952,333 raw particles were performed, leaving 168,307 particles in well-defined classes. These particles were used to generate ab initio models. These ab initio models were then used models were used in seven rounds of heterogenous refinement. This resulted in a class with 137,338 particles which had a resolution of 5.2 Å when refined with non-uniform refinement^44^. Local Refinement, with a generous mask around defined regions of density, was then used to generate a final map at 4.5 Å resolution, which was sharpened with DeepEMhancer^46^.

To obtain higher resolution detail for the leptin-LepR binding interface, a mask was generated around leptin and each domain of the LepR complex which was contacting LepR. This was then used to perform focused, non-uniform, refinement on the 444,608 particles from the third iteration of the heterogenous refinements^47^. This resulted in a map which had a resolution of 3.8 Å, which was sharpened with cryoSPARC^48^.

### Model building and refinement

AlphaFold models^49^ of mouse leptin and LepR were docked into the various maps using UCSF Chimera^50^. The resultant model was then refined using Phenix real space refine^51^ and manual building in Coot^52^. The model for the full-length receptor had its sidechains truncated to Cβ. Figures of cryo-EM maps and structural models were generated using UCSF ChimeraX^44^.

### Lentivirus production and lentiviral transduction

Lentiviruses were produced by transfection of HEK-293T cells (ATCC, CRL-3216) with pLV-EF1a-IRES-Puro vector containing an N-terminal Myc-tag in combination with the pMD2G envelope and psPax2 packaging plasmids. Forty-eight hours after transfection, the virus-containing supernatant was collected and centrifuged for 5 min at 300 × *g* to remove cell debris. Virus was concentrated by incubation with 1x PEG-IT (SBI) at 4 °C for 12–24 h. The solution was then centrifuged a 1500 × *g* for 30 min and virus pellet was resuspended with 10% of the initial virus volume in serum free DMEM. Target cells were plated in 6-well plates containing DMEM supplemented with 10% v/v fetal bovine serum, penicillin-streptomycin, 1 mM sodium pyruvate, 10 nM HEPES and 2 mM GlutaMAX^TM^ (Gibco). Concentrated virus was added to the media together with 8 µg/mL polybrene. Forty-eight hours later, the media was changed to fresh media containing puromycin for selection.

### Cell signaling assays

For analysis of human LepR mutants, HEK-293T cells were plated in six-well culture dishes coated with fibronectin (Millipore) at 0.7 × 10^6^ cells per well in DMEM (Dulbecco’s Modified Eagle Medium) supplemented with 10% v/v fetal bovine serum, penicillin-streptomycin, 1 mM sodium pyruvate, 10 nM HEPES and 2 mM GlutaMAX^TM^ (Gibco). Forty-eight hours later, cells were transfected using FuGene 6 (Promega) with pD649 vector containing HA- or FLAG-tagged full-length human LepR, WT or mutant. Twenty-four hours after transfection, cells were treated with recombinant human Leptin for 20 min at 37 °C. Cells were then rinsed once with ice-cold PBS and immediately lysed with Triton lysis buffer (1% v/v Triton, 20 mM HEPES pH 7.4, 150 mM NaCl, one tablet of PhosSTOP phosphatase inhibitor cocktail (Roche), and one tablet of EDTA-free protease inhibitor (Roche) (per 10 ml buffer). The cell lysates were cleared by centrifugation at 15,000 × *g* at 4 °C for 10 min. Cell lysates were denatured by the addition of SDS sample buffer and boiling for 5 min., resolved by SDS-PAGE, and analyzed by immunoblotting.

For immunoblot-based analysis of human Leptin variants, HEK-293T cells stably expressing Myc-tagged full-length human LepR were plated in six-well culture dishes coated with fibronectin (Millipore) at 1 × 10^6^ cells per well in DMEM (Dulbecco’s Modified Eagle Medium) supplemented with 10% v/v fetal bovine serum, penicillin-streptomycin, 1 mM sodium pyruvate, 10 nM HEPES and 2 mM GlutaMAX^TM^ (Gibco). Twenty-Four hours later, cells were treated with recombinant human Leptin for 20 min at 37 °C. Cells were then rinsed one time with ice-cold PBS and immediately lysed and analyzed as described above. For experiments analyzing phosphorylation of ERK and SHP2, 22 h after transfection cell media was replaced with serum free DMEM for an additional 24 h before addition of recombinant Leptin. Antibodies used for immunoblots were obtained from Cell Signaling Technologies and include: Phospho-STAT3 (Y705, Antibody #9131, 1:1000), Phospho-SHP2 (Y542, Antibody #3751, 1:1000), Phospho-ERK1/2 (T202/Y204, Antibody #9101, 1:3000), STAT3 (clone 79D7, 1:1000), SHP2 (Antibody #3752, 1:1000), ERK1/2 (clone 137F5, 1:5000), HA (clone C29F4, 1:5000). Signal intensity for immunoblot experiments were quantified using ImageJ v10.2. Uncropped immunoblot images are presented in Supplementary Fig. 9.

For flow-cytometry-based signaling experiments, HEK-293T cells stably expressing Myc-tagged full-length human LepR were plated in 96-well plates and stimulated with WT or mutant Leptin for 20 min at 37 °C, followed by fixation with paraformaldehyde (Electron Microscopy Sciences) for 10 min at room temperature. The cells were permeabilized for intracellular staining by treatment with ice-cold methanol (Fisher) for 30 min at −20 °C. The cells were then incubated with Alexa Fluor 647 conjugated Anti-Stat3 (pY705) antibody (1:100, BD, clone 4/P-STAT3) and anti-c-Myc-Alexa Fluor 488 (1:100, CST, clone 9B11) for 1 h at room temperature in autoMACS buffer (Miltenyi). Data were acquired using CytoFlex, flow cytometer instrument (Beckman Coulter). The MFI values were background subtracted and represented as a percent of the maximal WT Leptin value within each experiment and plotted in Prism 8 (GraphPad). The dose-response curves were generated using the “sigmoidal dose-response” analysis.

### Gene expression analysis

For gene expression analysis by qPCR, HEK-293T cells stably expressing Myc-tagged full-length human LepR were treated with PBS or Leptin variants for 5 h. Cells were isolated and lysed using Qiashredder columns (Qiagen) per the manufacturer’s instructions. RNA was isolated using RNAeasy plus mini kit (Qiagen) per the manufacturer’s instructions. One microgram RNA for each sample was used for cDNA generation with iScript Reverse Transcription Supermix (BioRad). Relative gene expression was measured by SYBR-green based qPCR using the comparative ΔC_t_ method and normalized to *GAPDH* expression. All samples were run in triplicate. The following mouse qPCR primers were obtained from IDT: *GAPDH:* (5′GGA AAC TTG CTG TGG GTG A3′, 5′CAA GGA CGG AGA CTT CGA TTC3′), *SOCS3:* (5′TGT AGT TGA GGT CAA TGA AGG G3′, 5′ACA TCG CTC AGA CAC CAT G3′).

### In vivo studies

Animal experiments were performed according to a procedure approved by the Stanford University Administrative Panel on Laboratory Animal Care (APLAC). Mice were maintained in 12-h light–dark cycles at 22 °C and ~50% relative humidity and fed a standard irradiated rodent chow diet. Male B6.Cg-*Lep*^*ob*^/J (stock no. 000632) were purchased from Jackson Laboratory. Proteins were diluted in sterile PBS and were administered twice a day at 10 am and 6 pm via intraperitoneal injection at 2.5 μL/g body weight at the indicated doses. Mice were mock injected with PBS 5 days before the experiment until the body weights were stabilized.

### Reporting summary

Further information on research design is available in the Nature Portfolio Reporting Summary linked to this article.

## Supplementary information

Supplementary Information

Peer Review File

Reporting Summary

## Data Availability

The data that support this study are available from the corresponding authors upon request. Cryo-EM maps have been deposited in the Electron Microscopy Data Bank 9EMDB under accession codes EMD-27432 (LepR^D1–^^D7^ receptor complex), EMD-27433 (LepR^D3–^^D7^ receptor complex), and EMD-27434 (LepR focused interaction). Atomic coordinates have been deposited in the Protein Data Bank (PDB) under accession codes 8DH8 (LepR^D1–^^D7^ receptor complex), 8DH9 (LepR^D3–D7^ receptor complex), and 8DHA (LepR focused interaction). Source data underling Fig. 4b, d, f, g, i, and Supplementary Fig. 8b–e are provided in the Source Data File. Source data are provided with this paper.

## Source data

Source Data

## References

[1] Blüher M (2019). Obesity: global epidemiology and pathogenesis. Nat. Rev. Endocrinol..

[2] Hruby A, Hu FB (2015). The epidemiology of obesity: a big picture. Pharmacoeconomics.

[3] Müller TD, Blüher M, Tschöp MH, DiMarchi RD (2022). Anti-obesity drug discovery: advances and challenges. Nat. Rev. Drug Discov..

[4] Zhang Y (1994). Positional cloning of the mouse obese gene and its human homologue. Nature.

[5] Friedman JM (2019). Leptin and the endocrine control of energy balance. Nat. Metab..

[6] Montague CT (1997). Congenital leptin deficiency is associated with severe early-onset obesity in humans. Nature.

[7] Halaas JL (1995). Weight-reducing effects of the plasma protein encoded by the obese gene. Science.

[8] Pelleymounter MA (1995). Effects of the obese gene product on body weight regulation in ob/ob mice. Science.

[9] Farooqi IS (1999). Effects of recombinant leptin therapy in a child with congenital leptin deficiency. N. Engl. J. Med..

[10] Heymsfield SB (1999). Recombinant leptin for weight loss in obese and lean adults: a randomized, controlled, dose-escalation trial. JAMA.

[11] Frederich RC (1995). Leptin levels reflect body lipid content in mice: evidence for diet-induced resistance to leptin action. Nat. Med..

[12] Enriori PJ (2007). Diet-induced obesity causes severe but reversible leptin resistance in arcuate melanocortin neurons. Cell Metab..

[13] Allison MB, Myers MG (2014). 20 years of leptin: connecting leptin signaling to biological function. J. Endocrinol..

[14] Ahima RS, Flier JS (2000). Leptin. Annu. Rev. Physiol..

[15] Mori H (2004). Socs3 deficiency in the brain elevates leptin sensitivity and confers resistance to diet-induced obesity. Nat. Med..

[16] Bjørbaek C, Elmquist JK, Frantz JD, Shoelson SE, Flier JS (1998). Identification of SOCS-3 as a potential mediator of central leptin resistance. Mol. Cell.

[17] Knight ZA, Hannan KS, Greenberg ML, Friedman JM (2010). Hyperleptinemia is required for the development of leptin resistance. PLoS ONE.

[18] Bjørbaek C, El-Haschimi K, Frantz JD, Flier JS (1999). The role of SOCS-3 in leptin signaling and leptin resistance. J. Biol. Chem..

[19] Myers MG, Cowley MA, Münzberg H (2008). Mechanisms of leptin action and leptin resistance. Annu. Rev. Physiol..

[20] Zhao S (2019). Partial leptin reduction as an insulin sensitization and weight loss strategy. Cell Metab..

[21] Funcke JB (2014). Monogenic forms of childhood obesity due to mutations in the leptin gene. Mol. Cell Pediatr..

[22] Farooqi IS (2007). Clinical and molecular genetic spectrum of congenital deficiency of the leptin receptor. N. Engl. J. Med..

[23] Huvenne H (2015). Seven novel deleterious LEPR mutations found in early-onset obesity: a ΔExon6-8 shared by subjects from Reunion Island, France, suggests a founder effect. J. Clin. Endocrinol. Metab..

[24] Howard JK (2004). Enhanced leptin sensitivity and attenuation of diet-induced obesity in mice with haploinsufficiency of Socs3. Nat. Med..

[25] Bjorbak C (2000). SOCS3 mediates feedback inhibition of the leptin receptor via Tyr985. J. Biol. Chem..

[26] Bates SH (2003). STAT3 signalling is required for leptin regulation of energy balance but not reproduction. Nature.

[27] Kievit P (2006). Enhanced leptin sensitivity and improved glucose homeostasis in mice lacking suppressor of cytokine signaling-3 in POMC-expressing cells. Cell Metab..

[28] Iserentant H (2005). Mapping of the interface between leptin and the leptin receptor CRH2 domain. J. Cell Sci..

[29] Peelman F (2004). Mapping of the leptin binding sites and design of a leptin antagonist. J. Biol. Chem..

[30] Bates SH, Kulkarni RN, Seifert M, Myers MG (2005). Roles for leptin receptor/STAT3-dependent and -independent signals in the regulation of glucose homeostasis. Cell Metab..

[31] Björnholm M (2007). Mice lacking inhibitory leptin receptor signals are lean with normal endocrine function. J. Clin. Investig..

[32] Saxton RA (2021). The tissue protective functions of interleukin-22 can be decoupled from pro-inflammatory actions through structure-based design. Immunity.

[33] Maffei M (1995). Leptin levels in human and rodent: measurement of plasma leptin and ob RNA in obese and weight-reduced subjects. Nat. Med..

[34] Considine RV (1996). Serum immunoreactive-leptin concentrations in normal-weight and obese humans. N. Engl. J. Med..

[35] Shpilman M (2011). Development and characterization of high affinity leptins and leptin antagonists. J. Biol. Chem..

[36] Mitra S (2015). Interleukin-2 activity can be fine tuned with engineered receptor signaling clamps. Immunity.

[37] Scapin G (2018). Structure of the insulin receptor-insulin complex by single-particle cryo-EM analysis. Nature.

[38] Uchikawa, E., Choi, E., Shang, G., Yu, H. & Bai, X. C. Activation mechanism of the insulin receptor revealed by cryo-EM structure of the fully liganded receptor-ligand complex. *Elife* **8**. 10.7554/eLife.48630 (2019). 10.7554/eLife.48630 PMC6721835 31436533

[39] Alvarado D, Klein DE, Lemmon MA (2010). Structural basis for negative cooperativity in growth factor binding to an EGF receptor. Cell.

[40] Tsirigotaki, A. et al. Mechanism of receptor assembly via the pleiotropic adipokine Leptin. *Nat Struct Mol Biol*10.1038/s41594-023-00941-9 (2023). 10.1038/s41594-023-00941-9 36959263

[41] Wilmes S (2020). Mechanism of homodimeric cytokine receptor activation and dysregulation by oncogenic mutations. Science.

[42] Glassman CR (2022). Structure of a Janus kinase cytokine receptor complex reveals the basis for dimeric activation. Science.

[43] Tao P (2020). Selection of a full agonist combinatorial antibody that rescues leptin deficiency in vivo. Adv. Sci..

[44] Pettersen EF (2021). UCSF ChimeraX: structure visualization for researchers, educators, and developers. Protein Sci..

[45] Ramlaul K, Palmer CM, Aylett CHS (2019). A local agreement filtering algorithm for transmission EM reconstructions. J. Struct. Biol..

[46] Sanchez-Garcia R (2021). DeepEMhancer: a deep learning solution for cryo-EM volume post-processing. Commun. Biol..

[47] Punjani A, Zhang H, Fleet DJ (2020). Non-uniform refinement: adaptive regularization improves single-particle cryo-EM reconstruction. Nat. Methods.

[48] Punjani A, Rubinstein JL, Fleet DJ, Brubaker M (2017). A. cryoSPARC: algorithms for rapid unsupervised cryo-EM structure determination. Nat. Methods.

[49] Tunyasuvunakool K (2021). Highly accurate protein structure prediction for the human proteome. Nature.

[50] Pettersen EF (2004). UCSF Chimera-a visualization system for exploratory research and analysis. J. Comput. Chem..

[51] Adams PD (2010). PHENIX: a comprehensive Python-based system for macromolecular structure solution. Acta Crystallogr. D Biol. Crystallogr..

[52] Emsley P, Cowtan K (2004). Coot: model-building tools for molecular graphics. Acta Crystallogr. D Biol. Crystallogr..

