# Supplementary material for: Structural insights into the mechanism of leptin receptor activation
Source: Nat Commun. 2023 Mar 31;14:1797. doi: 10.1038/s41467-023-37169-6 (PMC10066393; doi:10.1038/s41467-023-37169-6)
Supplement: Supplementary file 1 — Supplementary Information [file 41467_2023_37169_MOESM1_ESM.pdf]

Supplementary Materials for

Structural insights into the mechanism of leptin receptor activation

Robert A. Saxton[1,2,*,†], Nathanael A. Caveney[1,*], Maria Dolores Moya-Garzon[3], Karsten D. Householder[1], Grayson E. Rodriguez[1,4], Kylie A. Burdsall[5], Jonathan Z. Long[3], and K. Christopher Garcia[1,6,7,†]

Supplementary Table 1. Cryo-EM data collection refinement, and validation statistics.

	Leptin–LepR^{D1-D7} complex (PDB 8DH8/EMD-27432)	Leptin–LepR^{D3-D7} complex (PDB 8DH9/EMD-27433)	Leptin–LepR^{D1-D7} complex – focused refinement (PDB 8DHA /EMD-27434)
**Data collection and processing**			
Voltage (keV)	300	300	
Electron exposure ($e^-/Å^2$)	53	53	
Defocus range (μm)	-0.8 to -2.0	-0.8 to -2.0	
Pixel size (Å)	0.653	0.8521	
Symmetry imposed	C1	C1	
Initial particle images	3,150,756	17,952,333	
Final particle images	270,721	137,338	460,818
Map resolution FSC threshold (Å)	0.143	0.143	0.143
Map resolution (Å)	5.9	4.5	3.8
**Refinement**			
Initial model used (PDB)	AlphaFold	AlphaFold	
Model resolution FSC threshold (Å)	0.5	0.5	0.5
Model resolution (Å)	8.1	4.3	4.3
Map sharpening *B*-factor ($Å^2$)	288.6	118.5	150.0
Model Composition			
Non-hydrogen atoms	7,644	10,229	3,510
Protein residues	1,541	1,281	439
Ligands	-	-	2
B-factors ($Å^2$)			
Protein	477.59	186.15	204.60
Ligand	-	-	180.15
R.m.s. deviations			
Bond lengths (Å)	0.005	0.003	0.004
Bond angles (°)	1.157	0.649	0.789
Validation			
MolProbity score	1.96	1.84	2.00
Clashscore	13.09	13.96	14.65
Rotamer outliers (%)	-	0.08	0.49
Ramachandran plot			
Favoured (%)	95.17	93.86	95.15
Allowed (%)	4.63	2.91	4.85
Outliers (%)	0.20	0.24	0.00

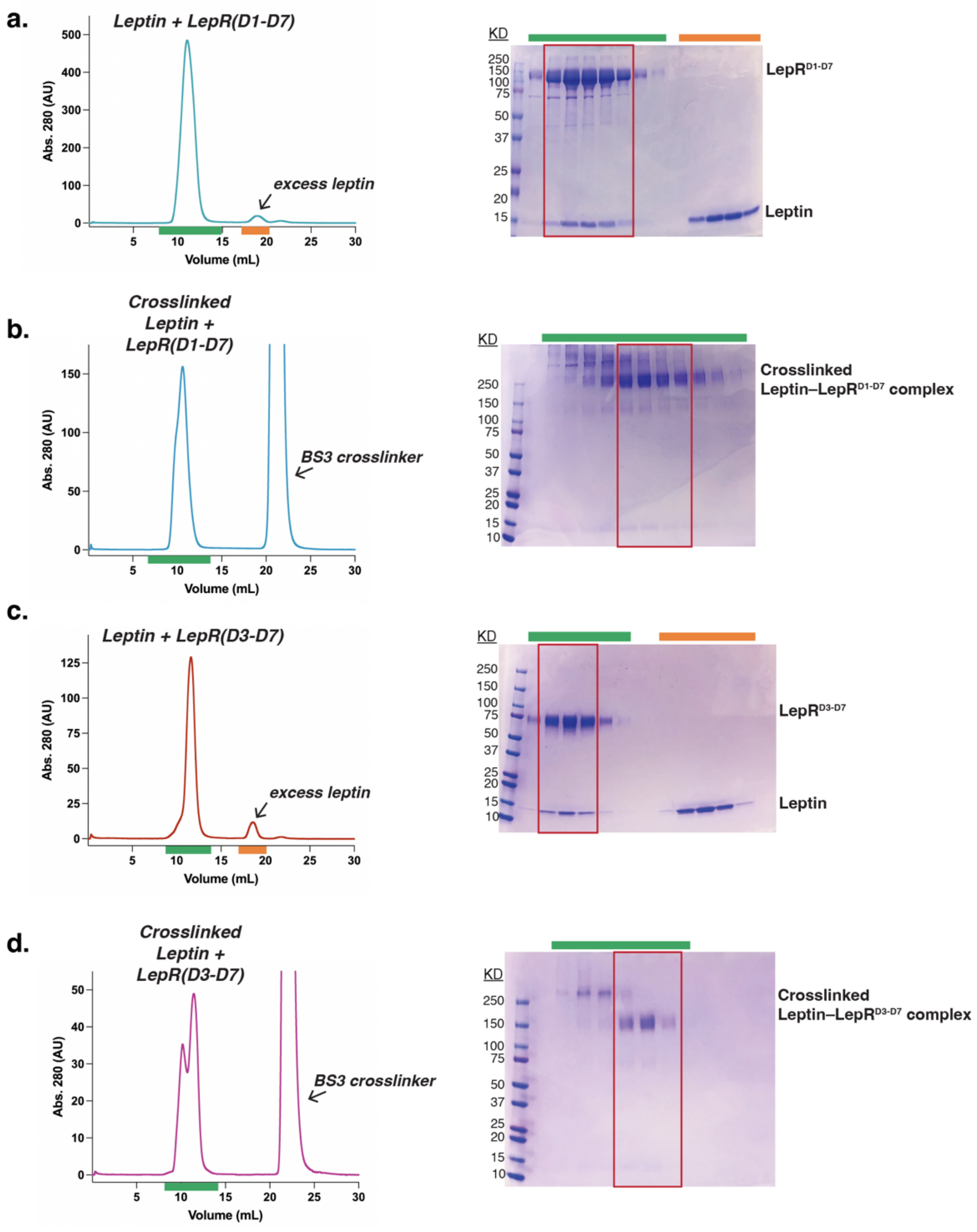

Supplementary Figure 1: Assembly and purification of the mouse leptin receptor complex. (**a-d**) Size-exclusion chromatography profiles and corresponding SDS-PAGE gels of the assembled Leptin-LepR^{D1-D7} complex (**a**), crosslinked Leptin-LepR^{D1-D7} complex (**b**), Leptin-LepR^{D3-D7} complex (**c**), and crosslinked Leptin-LepR^{D3-D7} complex (d). Green bars indicate the Leptin–LepR complex, orange bars indicate free Leptin, and red box indicates fractions that were collected for further processing and structural characterization.

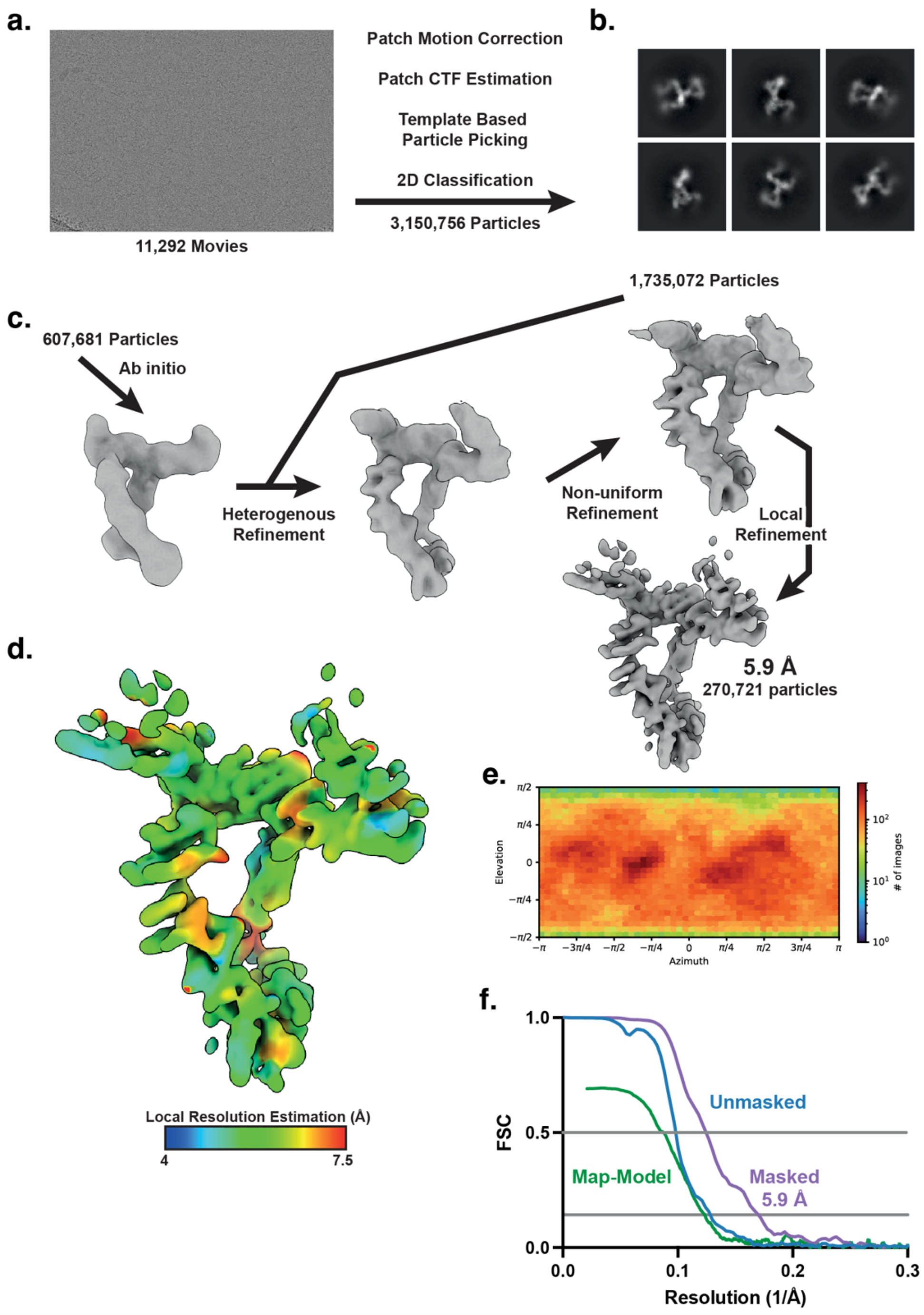

Supplementary Figure 2: Leptin–LepR$^{D1\text{-}D7}$ complex cryo-EM data processing. (**a**) Representative cryo-EM micrograph of the leptin–LepR$^{D1\text{-}D7}$ complex. (**b**) Reference free 2D class averages of the Leptin–LepR$^{D1\text{-}D7}$ complex. (**c**) Workflow for processing Leptin–LepR$^{D1\text{-}D7}$ complex cryo-EM dataset. (**d**) Local resolution of the Leptin–LepR$^{D1\text{-}D7}$ complex cryo-EM density map, ranging from 4.0 Å to 7.5 Å resolution. (**e**) Euler angle orientation distribution for Leptin–LepR$^{D1\text{-}D7}$ complex cryo-EM dataset. (**f**) Half-set gold-standard FSC and map vs model FSC curves for Leptin–LepR$^{D1\text{-}D7}$ complex.

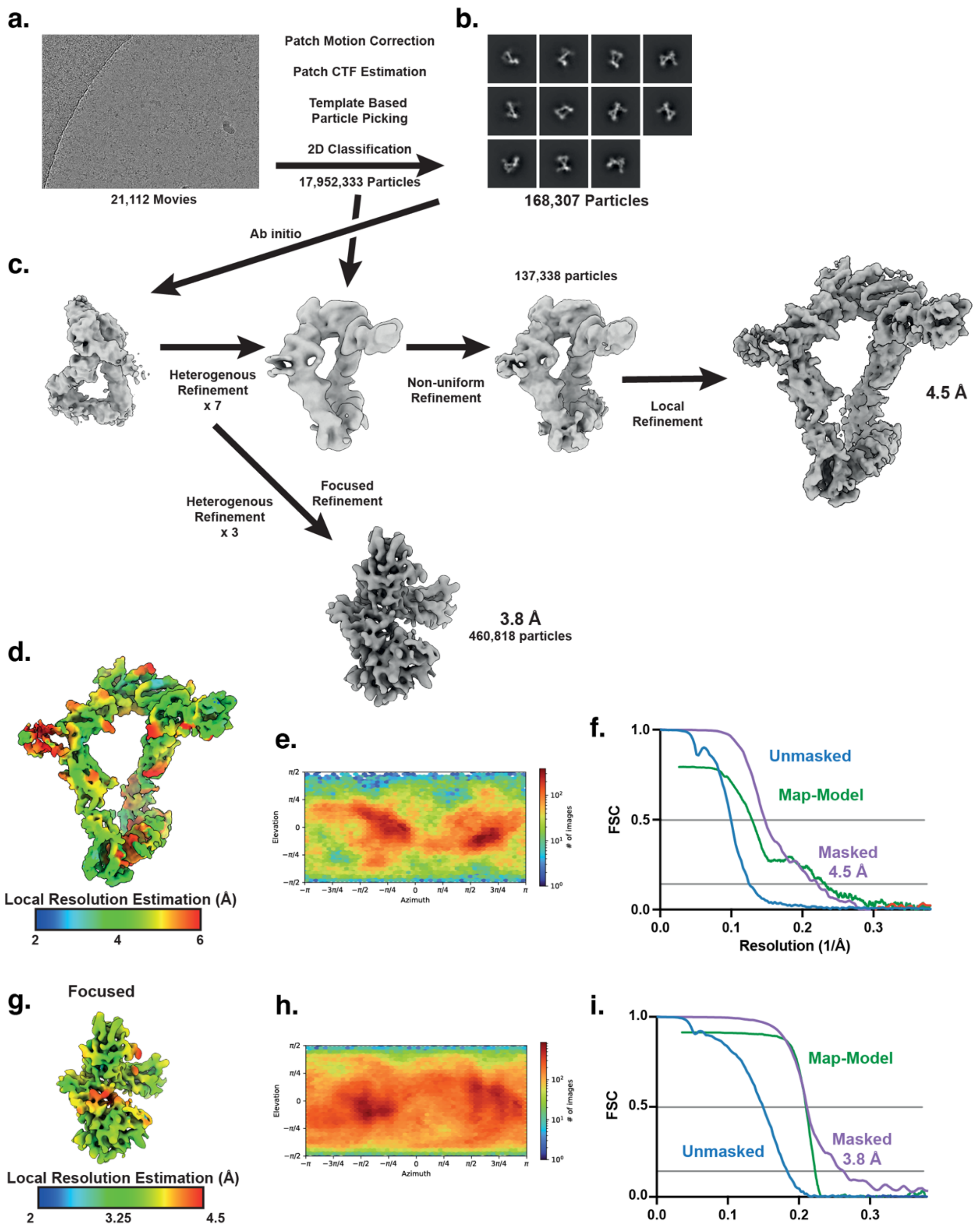

Supplementary Figure 3: Leptin–LepR^{D3-D7} complex cryo-EM data processing. (**a**) Representative cryo-EM micrograph of the Leptin–LepR^{D3-D7} complex. (**b**) 2D class averages of the Leptin–LepR^{D3-D7} complex. (**c**) Workflow for processing Leptin–LepR^{D3-D7} complex cryo-EM dataset. (**d**) Local resolution of the Leptin–LepR^{D3-D7} complex cryo-EM density map, ranging from 2.0 Å to 6.0 Å resolution. (**e**) Euler angle orientation distribution for Leptin–LepR^{D3-D7} complex cryo-EM dataset. (**f**) Half-set gold-standard FSC and map vs model FSC curves for Leptin–LepR^{D3-D7} complex. (**g**) Local resolution cryo-EM density map of the focus refined region of the Leptin–LepR^{D3-D7} complex, ranging from 2.0 Å to 4.5 Å resolution. (**h**) Euler angle orientation distribution for the focus refined region of Leptin–LepR^{D3-D7} complex cryo-EM dataset. (**i**) Half-set gold-standard FSC and map vs model FSC curves for the focus refined region of Leptin–LepR^{D3-D7} complex.

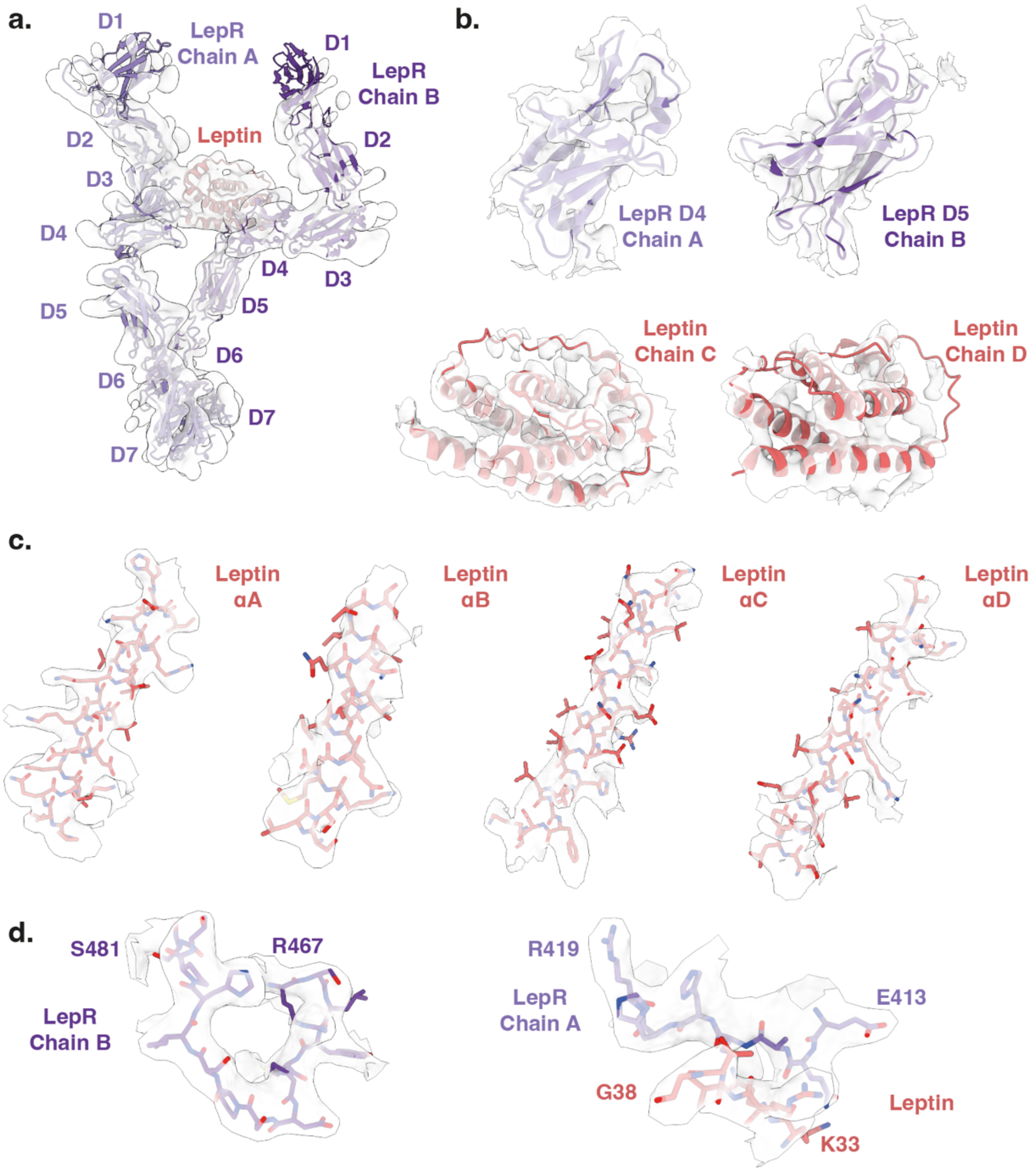

Supplementary Figure 4: Analysis of cryo-EM map quality. (**a**) Molecular model of the leptin-LepR$^{D1\text{-}D7}$ complex fit into its 5.9 Å resolution cryo-EM density map. (**b**) Sample cryo-EM density map and fitted molecular model for key regions of the 4.5 Å resolution cryo-EM density map of the leptin–LepR$^{D3\text{-}D7}$ complex. (**c,d**) Sample cryo-EM density map and fitted molecular model for key regions at the focus refine d 3.8 Å resolution map of the leptin–LepR interface, showing that secondary structure and bulky side chain s can be resolved at the current resolution.

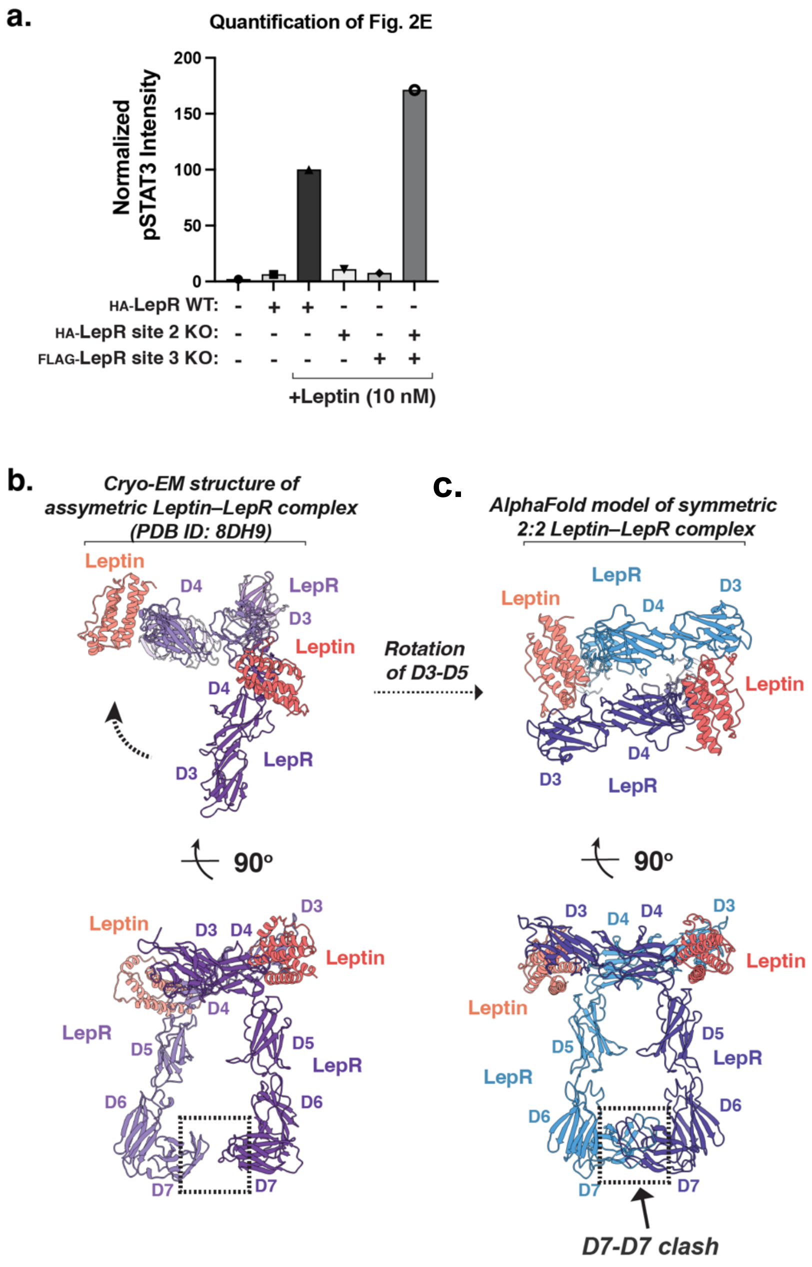

Supplementary Figure 5: Analysis of “open” LepR complex. (**a**) Quantification of immunoblot in Figure 2e. (**b**) Top and side views for the Leptin–LepR$^{D3\text{-}D7}$ complex reported here, highlighting the asymmetric, “open” conformation of the one LepR D3 domain, and absence of steric hindrance at the membrane proximal D7 domains. (**c**) Top and side view of Leptin–LepR model in which the second LepR D3 domain is closed to form a site 3 contact with the second bound Leptin, analogous the symmetric receptor complex observed in the IL-6 receptor complex structure. This rotation results in a significant steric clash between the membrane proximal D7 domains of the two LepR chains.

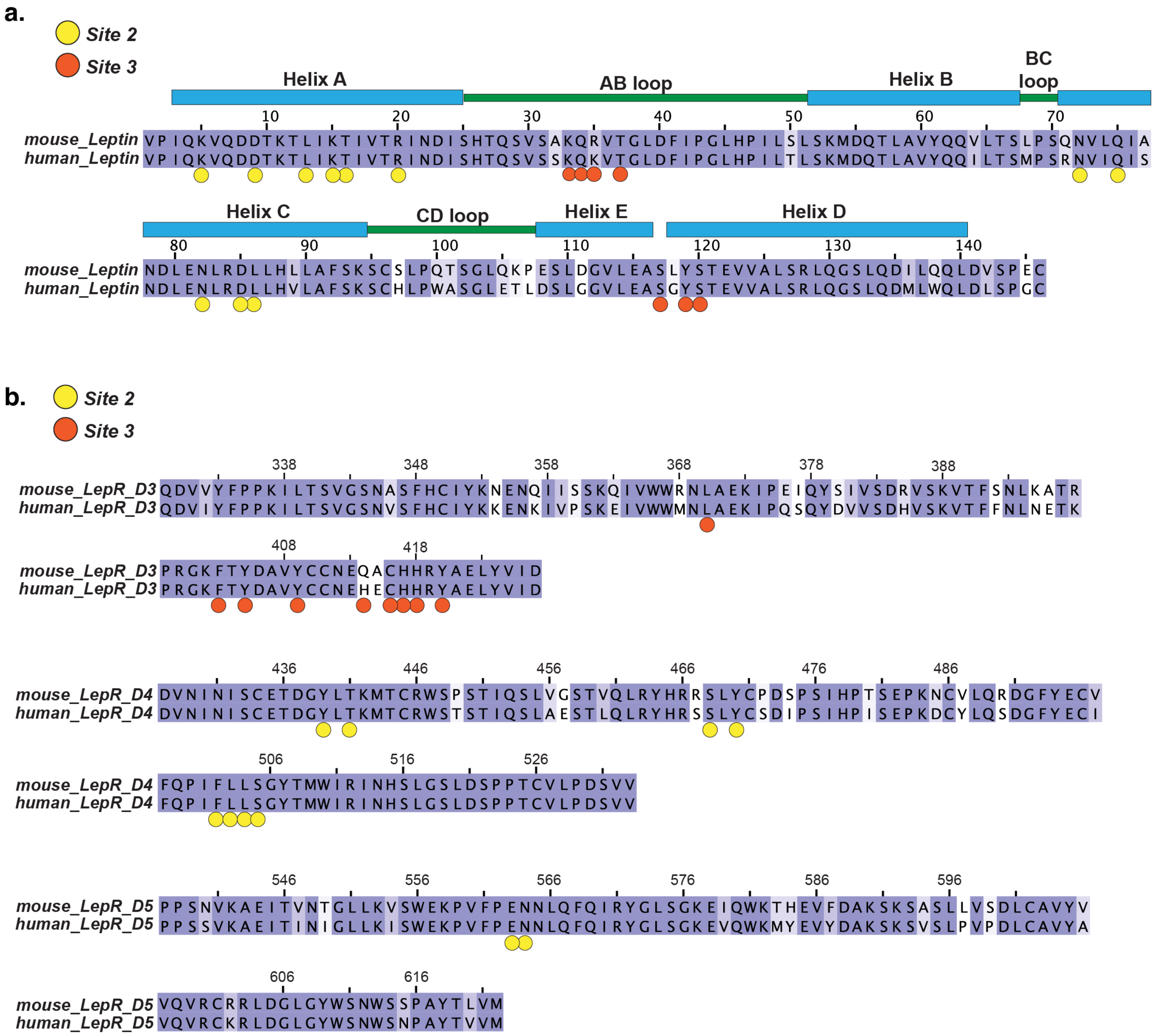

Supplementary Figure 6: Conservation of the Leptin–LepR binding interface. (**a**) Sequence alignment of mouse and human Leptin with positions colored from white to blue according to increasing sequence identity. Residues involved in LepR binding at site 2 (yellow) and site 3 (orange) are indicated. (**b**) Sequence alignment of the Leptin binding domains of mouse and human LepR (D3, D4, and D5) with positions colored from white to blue according to increasing sequence identity. Residues involved in Leptin binding at site 2 (yellow) and site 3 (orange) are indicated.

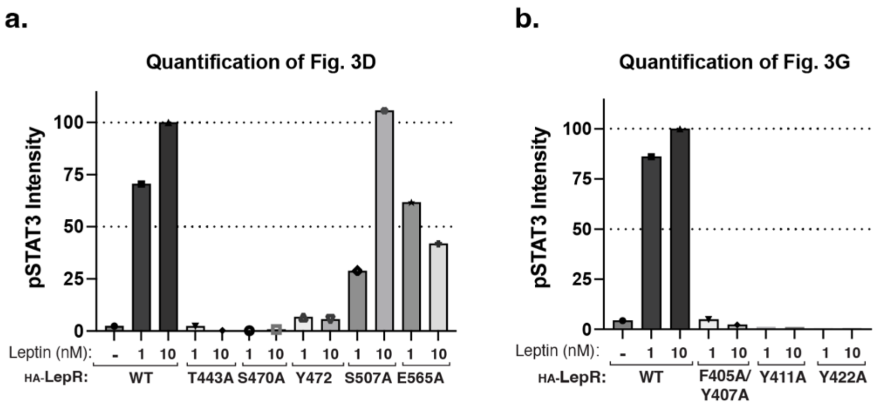

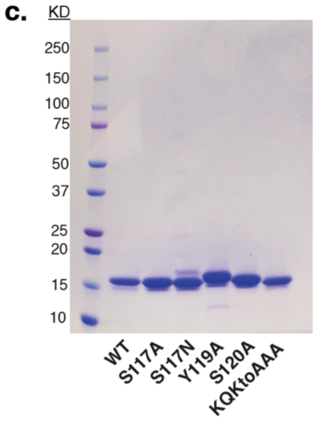

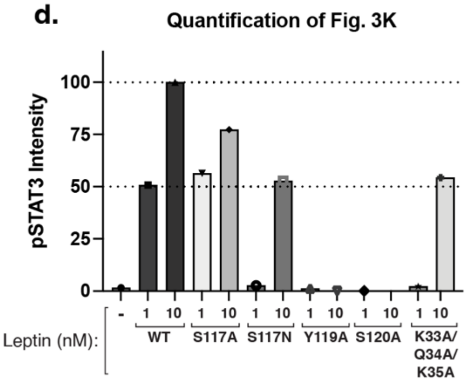

e.

	293T-cells expressing hLepR	
	EC_{50} (nM)	E_{max} (%)
**WT**	2.2	100.7 ± 1.7
**K33A/Q34A**	2.2	95.5 ± 3.1
**K33T/Q34T**	2.9	93.2 ± 2.0
**K35A**	4.1	89.9 ± 3.2
**K33A/Q34A /K35A**	3.9	73.5 ± 1.8
**S117A**	2.8	88.1 ± 1.6
**S117N**	8.1	43.0 ± 1.0
**Y119A**	18.0	8.0 ± 1.1
**S120A**	nd	nd

Supplemnentary Figure 7: Functional Characterization of Leptin and LepR mutants. (**a**, **b**) Quantification of immunoblots in Figure 3d (a) and 3g (b). (**C**) Coomassie-stained SDS-PAGE gel of recombinant leptin variants. (**c**) Coomassie-stained SDS-PAGE gel of recombinant leptin variants. (**d**) Quantification of immunoblots from Figure 3k. (**d**) Quantification of immunoblots from Figure 3K. (**e**) Table of calculated EC_{50} and E_{max} values for all leptin mutants tested on mLepR expressing 293T cells (nd, not determined).

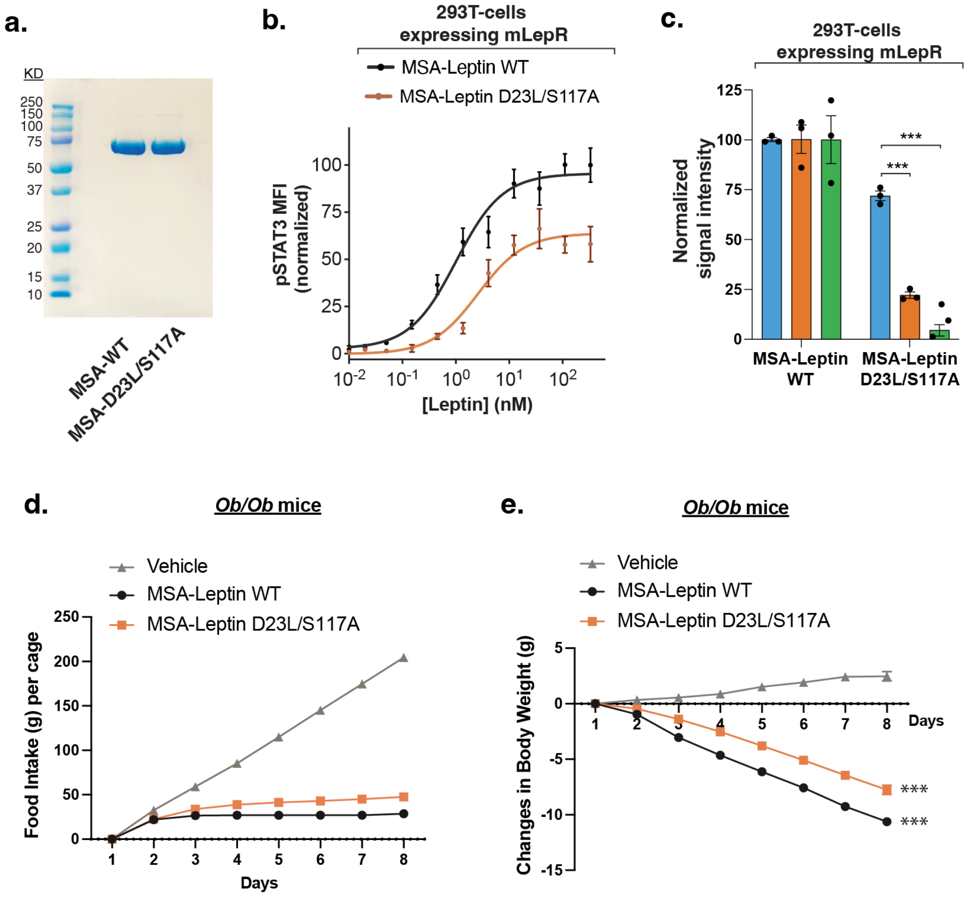

Supplementary Figure 8: Characterization of a biased mouse LepR analog. (**a**) Coomassie-stained SDS-PAGE gel of recombinant mouse serum albumin (MSA)-conjugated leptin variants. (**b**) Phospho-STAT3 dose-response curves for WT or mutant leptin in mouse LepR-expressing HEK 293T cells, analyzed as in (mean ± SEM, n=3 independent replicates). (**C**) Quantification of immunoblots of lysates prepared from HEK-293T cells stably expressing wild-type mouse LepR that were serum starved for 18 hours and then stimulated with 10 nM leptin (mean ± SEM, n=3, two-sided t-test, ****P*<0.001). (**d, E**) Eight-week-old B6.Cg-*Lep*^{ob}/J mice were treated with either vehicle (PBS) or 80 µg of MSA-conjugated leptin variant D23L/S117A twice daily via I.P. injection for seven days (mean ± SEM, n=5 mice/group, two-way ANOVA, ****P*<0.001).

a. Corresponding to Figure 2e:

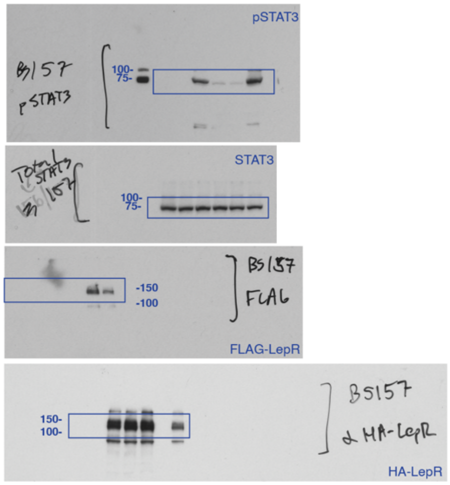

c. Corresponding to Figure 3g:

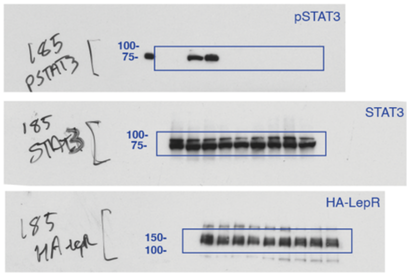

f. Corresponding to Figure 4h:

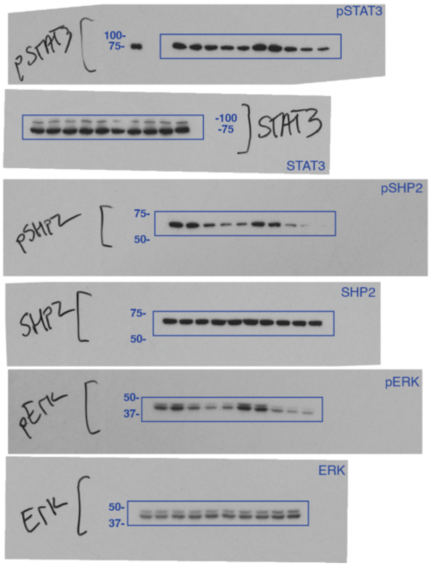

b. Corresponding to Figure 3d:

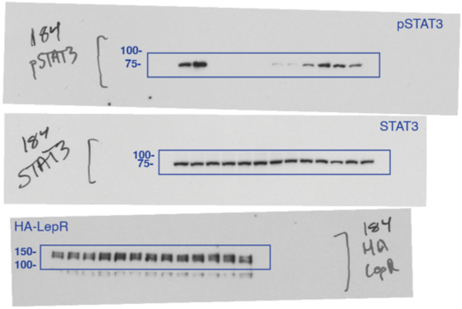

d. Corresponding to Figure 3k:

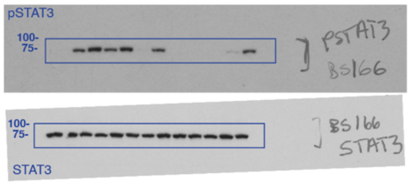

e. Corresponding to Figure 4c:

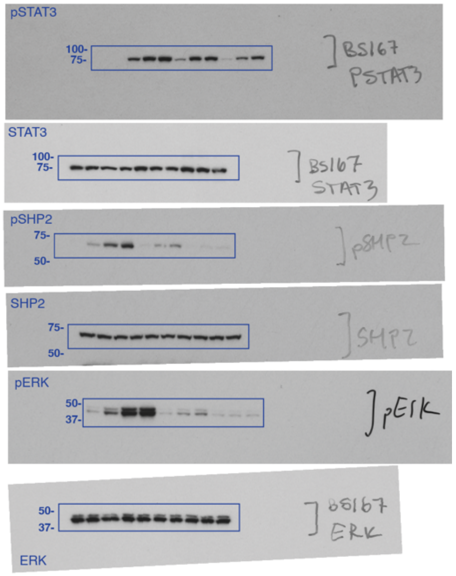

Supplementary Figure 9: Raw scans of immunoblots. (**a-f**) Raw scans of immunoblots used in the corresponding figures. Blots of unphosphorylated proteins were used as sample processing controls and run on separate gels. Blue square indicates region of blots used in final figure.
