# Supplementary material for: Structural insights into the mechanism of leptin receptor activation
Source: Nat Commun. 2023 Mar 31;14:1797. doi: 10.1038/s41467-023-37169-6 (PMC10066393; doi:10.1038/s41467-023-37169-6)
Supplement: Supplementary file 3 — Reporting Summary [file 41467_2023_37169_MOESM3_ESM.pdf]

nature portfolio

Corresponding author(s): K. Christopher Garcia
Robert A. Saxton

Last updated by author(s): 2/16/2023

Reporting Summary

Nature Portfolio wishes to improve the reproducibility of the work that we publish. This form provides structure for consistency and transparency in reporting. For further information on Nature Portfolio policies, see our Editorial Policies and the Editorial Policy Checklist.

Statistics

For all statistical analyses, confirm that the following items are present in the figure legend, table legend, main text, or Methods section.

n/a	Confirmed	
☐	☒	The exact sample size (*n*) for each experimental group/condition, given as a discrete number and unit of measurement
☐	☒	A statement on whether measurements were taken from distinct samples or whether the same sample was measured repeatedly
☐	☒	The statistical test(s) used AND whether they are one- or two-sided<br>*Only common tests should be described solely by name; describe more complex techniques in the Methods section.*
☒	☐	A description of all covariates tested
☒	☐	A description of any assumptions or corrections, such as tests of normality and adjustment for multiple comparisons
☐	☒	A full description of the statistical parameters including central tendency (e.g. means) or other basic estimates (e.g. regression coefficient) AND variation (e.g. standard deviation) or associated estimates of uncertainty (e.g. confidence intervals)
☐	☒	For null hypothesis testing, the test statistic (e.g. *F*, *t*, *r*) with confidence intervals, effect sizes, degrees of freedom and *P* value noted<br>*Give P values as exact values whenever suitable.*
☒	☐	For Bayesian analysis, information on the choice of priors and Markov chain Monte Carlo settings
☒	☐	For hierarchical and complex designs, identification of the appropriate level for tests and full reporting of outcomes
☒	☐	Estimates of effect sizes (e.g. Cohen's *d*, Pearson's *r*), indicating how they were calculated

Our web collection on statistics for biologists contains articles on many of the points above.

Software and code

Policy information about availability of computer code

Data collection	SerialEM
Data analysis	cryoSPARC v3.1.0 , LAFTER, UCSF ChimeraX v1.3, Phenix v1.19.2, Coot v0.9.4.1, Prism 8, Image J v1.53t

For manuscripts utilizing custom algorithms or software that are central to the research but not yet described in published literature, software must be made available to editors and reviewers. We strongly encourage code deposition in a community repository (e.g. GitHub). See the Nature Portfolio guidelines for submitting code & software for further information.

Data

Policy information about availability of data

All manuscripts must include a data availability statement. This statement should provide the following information, where applicable:

- Accession codes, unique identifiers, or web links for publicly available datasets
- A description of any restrictions on data availability
- For clinical datasets or third party data, please ensure that the statement adheres to our policy

Cryo-EM maps and atomic coordinates for LepRD1-D7 receptor complex, LepRD3-D7 receptor complex, and the focused interaction have been deposited in the EMDB (EMD-27432, EMD-27433, EMD-27434) and PDB (8DH8, 8DH9, 8DHA) respectively.

Human research participants

Policy information about studies involving human research participants and Sex and Gender in Research.

Reporting on sex and gender	not applicable
Population characteristics	not applicable
Recruitment	not applicable
Ethics oversight	not applicable

Note that full information on the approval of the study protocol must also be provided in the manuscript.

Field-specific reporting

Please select the one below that is the best fit for your research. If you are not sure, read the appropriate sections before making your selection.

☒ Life sciences ☐ Behavioural & social sciences ☐ Ecological, evolutionary & environmental sciences

For a reference copy of the document with all sections, see nature.com/documents/nr-reporting-summary-flat.pdf

Life sciences study design

All studies must disclose on these points even when the disclosure is negative.

Sample size	Sample size was not predetermined but we performed experiments with group sizes based on existing published literature of similar experiments. For animal experiments, n =5 was chosen based on the previous publications in the field. Sizes of cryo-EM data sets were determined based on microscope availability and ability to generate maps of sufficient resolution.
Data exclusions	Low quality cryo-EM micrographs were removed using standard analysis software and protocols.
Replication	All experiments were performed at least two times with independent biological replicates, and replicates performed as indicated in Figure Legends.
Randomization	For animal studies. age and sex matched animals were assigned randomly to each experimental and control group where applicable.
Blinding	Blinding was not performed. In all experiments samples were prepared and analyzed by the same person, making blinding difficult.

Reporting for specific materials, systems and methods

We require information from authors about some types of materials, experimental systems and methods used in many studies. Here, indicate whether each material, system or method listed is relevant to your study. If you are not sure if a list item applies to your research, read the appropriate section before selecting a response.

Materials & experimental systems

n/a	Involved in the study	
☐	☒	Antibodies
☐	☒	Eukaryotic cell lines
☒	☐	Palaeontology and archaeology
☐	☒	Animals and other organisms
☒	☐	Clinical data
☒	☐	Dual use research of concern

Methods

n/a	Involved in the study	
☒	☐	ChIP-seq
☒	☐	Flow cytometry
☒	☐	MRI-based neuroimaging

Antibodies

Antibodies used	Alexa Fluor 647 conjugated Anti-Stat3 (pY705) antibody (1:100, BD, clone 4/P-STAT3, Cat#557815),<br>anti-c-Myc-Alexa Fluor 488 (1:100, CST, clone 9B11, Cat#2279S),<br>Phospho-STAT3 (Y705, Antibody #9131, Cell Signaling Technologies, Cat#9131),<br>Phospho-SHP2 (Y542, Antibody #3751, Cell Signaling Technologies, Cat#3751S),<br>Phospho-ERK1/2 (T202/Y204, Antibody #9101, Cell signaling Technologies, Cat#9101S),

STAT3 (clone 79D7, Cell Signaling Technologies, Cat#4904),
SHP2 (Antibody #3752, Cell Signaling Technologies, Cat#3752S),
ERK1/2 (clone 137F5, Cell Signaling Technologies, Cat#4695S),
HA (clone C29F4, Cell Signaling Technologies, Cat#3724)

Validation

Validation results for all antibodies are reported on the manufactruer's website and similar results were obtained in the publications indicated below.
Alexa Fluor 647 conjugated Anti-Stat3 (pY705) antibody (1:100, BD, clone 4/P-STAT3, Cat#557815), PMID:30314758
anti-c-Myc-Alexa Fluor 488 (1:100, CST, clone 9B11, Cat#2279S), PMID: 30889382
Phospho-STAT3 (Y705, Antibody #9131, Cell Signaling Technologies, Cat#9131), PMID: 32673566
Phospho-SHP2 (Y542, Antibody #3751, Cell Signaling Technologies, Cat#3751S), PMID: 31436532
Phospho-ERK1/2 (T202/Y204, Antibody #9101, Cell signaling Technologies, Cat#9101S), PMID: 27007855
STAT3 (clone 79D7, Cell Signaling Technologies, Cat#4904), PMID: 30205046
SHP2 (Antibody #3752, Cell Signaling Technologies, Cat#3752S), PMID: 30454647
ERK1/2 (clone 137F5, Cell Signaling Technologies, Cat#4695S), PMID: 27845624
HA (clone C29F4, Cell Signaling Technologies, Cat#3724) PMID: 28193319

Eukaryotic cell lines

Policy information about cell lines and Sex and Gender in Research

Cell line source(s)	HEK-293T (ATCC CRL-3216), Expi293F (Thermo)
Authentication	No cell line authentication was performed.
Mycoplasma contamination	HEK-293T cells tested negative for Mycoplasma. Expi293F cells were not tested for mycoplasma.
Commonly misidentified lines (See ICLAC register)	No commonly misidentified cell lines were used.

Animals and other research organisms

Policy information about studies involving animals; ARRIVE guidelines recommended for reporting animal research, and Sex and Gender in Research

Laboratory animals	Male B6.Cg-Lepob/J (stock no. 000632) werepurchased from Jackson Laboratory. Mice were maintained in 12-h light dark cycles at 22 °C and ~50% relative humidity and fed a standard irradiated rodent chow diet.
Wild animals	not applicable
Reporting on sex	Only male mice were analyzed in this study.
Field-collected samples	not applicable
Ethics oversight	Animal experiments were performed according to a procedure approved by the Stanford University Administrative Panel on Laboratory Animal Care (APLAC)

Note that full information on the approval of the study protocol must also be provided in the manuscript.
